# Activation of rapid oestrogen signalling in aggressive human breast cancers

**DOI:** 10.1002/emmm.201201615

**Published:** 2012-10-15

**Authors:** Coralie Poulard, Isabelle Treilleux, Emilie Lavergne, Katia Bouchekioua-Bouzaghou, Sophie Goddard-Léon, Sylvie Chabaud, Olivier Trédan, Laura Corbo, Muriel Le Romancer

**Affiliations:** 1 Université de LyonLyon, France; 2 Université Lyon 1Lyon, France; 3 Inserm U1052, Centre de Recherche en Cancérologie de LyonLyon, France; 4 CNRS UMR5286, Centre de Recherche en Cancérologie de LyonLyon, France; 5 Centre Léon BérardLyon, France; 6 Equipe LabelliséeLa Ligue, France; 7 Pathology Department, Centre Léon BérardLyon, France; 8 Biostatistics Unit, Centre Léon BérardLyon, France; 9 Department of Medical Oncology, Centre Léon BérardLyon, France

**Keywords:** arginine methylation, breast cancer, oestrogen receptors, PI3K, Src tyrosine kinase

## Abstract

Oestrogen receptors can mediate rapid activation of cytoplasmic signalling cascades by recruiting Src and PI3K. However, the involvement of this pathway in breast cancer remains poorly defined. We have previously shown that methylation of ERα is required for the formation of the ERα/Src/PI3K complex and that ERα is hypermethylated in a subset of breast cancers. Here, we used Proximity Ligation Assay to demonstrate that this complex is present in the cytoplasm of breast cancer cell lines as well as formalin-fixed, paraffin-embedded tumours. Of particular interest, the analysis of 175 breast tumours showed that overexpression of this complex in a subset of breast tumours correlates to the activation of the downstream effector Akt. Survival analysis revealed that high expression of this complex is an independent marker of poor prognosis and associated with reduced disease-free survival. Our data introduces the new concept that the rapid oestrogen pathway is operative *in vivo*. It also provides a rationale for patient stratification defined by the activation of this pathway and the identification of target therapies.

## INTRODUCTION

The existence of extranuclear steroid signalling has been known for almost 40 years (Pietras & Szego, 1977), although the molecular mechanisms involved still remain elusive. Oestrogen mediates its effects through ERα and ERβ, which function in the nucleus as ligand-dependent transcription factors and stimulate cell growth in various tissues, including breast epithelial cells (Mangelsdorf et al, 1995; McKenna & O'Malley, 2002; Tsai & O'Malley, 1994). Oestrogen receptor activity is also regulated by a plethora of post-translational modifications including phosphorylation, acetylation and methylation (Le Romancer et al, 2011). In addition, accumulating evidence indicates that oestrogens activate non-genomic pathways through a pool of conventional ERα located in the cytoplasm and/or at the plasma membrane (Hammes & Levin, 2007; Levin, 2009; Razandi et al, 2004). Although several partners for extranuclear ERα have been described in different cell types, the most conserved partners are PI3K and the tyrosine kinase Src (Castoria et al, 2001; Simoncini et al, 2000; Song et al, 2005). Besides this core complex, several adaptor scaffold proteins such as p130^Cas^ and MNAR (modulator of non-genomic activity of the oestrogen receptor) have also been found to be part of the complex (Barletta et al, 2004; Cabodi et al, 2004; Shupnik, 2004). After oestrogenic stimulation, the rapid formation of the protein complex triggers the activation of downstream signalling cascades involving the Ras/MAPK and Akt pathways (Castoria et al, 2001; Hammes & Levin, 2007). Mechanistically, we previously reported that methylation of ERα on arginine 260 by the arginine methyltransferase PRMT1 is a prerequisite for its association with Src, PI3K and the Focal Adhesion Kinase (FAK) as well as activation of its downstream effector Akt (Le Romancer et al, 2008, 2010). Using an antibody that specifically recognizes the methylated form of ERα (mERα), we have shown that this modification occurs in the cytoplasm of normal breast epithelial cells and is highly expressed in a subset of breast tumours (Le Romancer et al, 2008). Of note, the presence of mERα in the cytoplasm of tumour cells did not correlate with the clinical classification of ERα-positive or -negative tumours. This is because the population of ERα-positive cases included only tumours, which exhibited ERα nuclear staining. Our results thus suggested that oestrogen non-genomic signalling, which mirrors ERα methylation, occurs in normal breast tissue and could be deregulated in breast cancer. However, the existence of the oestrogen-mediated signalling complex remains a fundamental question to be clearly addressed.

For this purpose, we used Proximity Ligation Assay (PLA) technology to detect both ERα/PI3K and ERα/Src interactions in breast cancer specimens. Here, we show that the signalling complex is present in the cytoplasm of normal epithelial cells and highly expressed in some breast tumours. Of note, the amount of ERα/PI3K/Src correlates with both the level of ERα methylation and the activation of Akt, a crucial downstream target of this complex. Finally, we show that overexpression of the complex in a subset of invasive breast tumours is an independent marker of poor prognosis and associated with reduced disease-free survival (DFS). This opens new horizons for breast cancer treatment.

## RESULTS

### Detection of endogenous ERα/PI3K and ERα/Src interactions in human breast tumour cells

**Figure 1:**
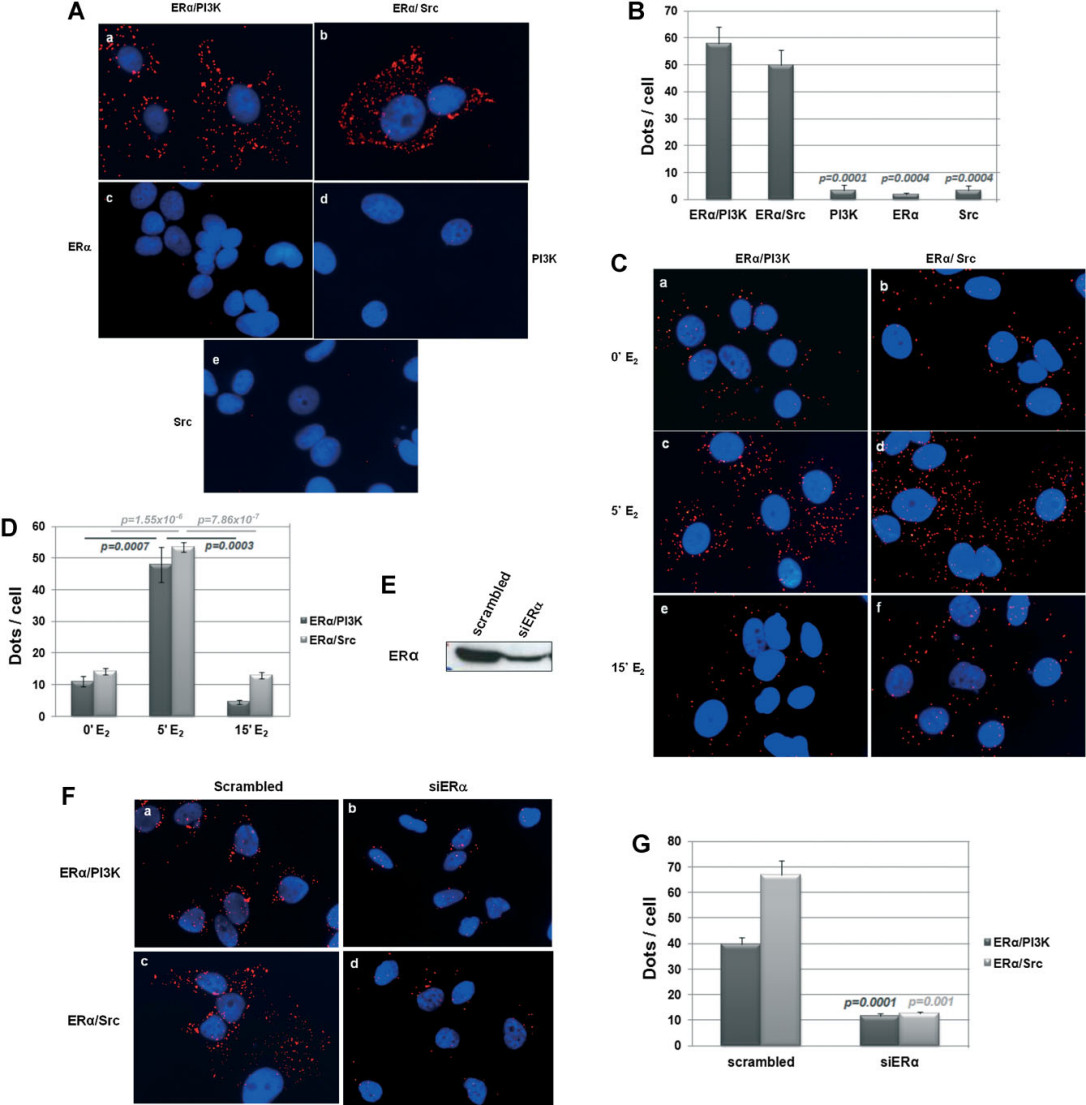
*In situ* PLA detection of endogenous ERα/PI3K and ERα/Src interactions in MCF-7 cells Oestrogen-deprived MCF-7 cells were incubated with E_2_ 10^−8^ M for 5 min. After fixation, *in situ* PLA for ERα/PI3K (panel a) and ERα/Src dimers (panel b) was performed with ERα-, Src- and PI3K-specific antibodies. The detected dimers are represented by red dots. The nuclei were counterstained with DAPI (blue) (Obj:X63). Control experiments were performed with single antibodies (panels c–e).Quantification of the number of signals per cell was performed by computer-assisted analysis as reported in the Materials and Methods Section. The mean ± SEM of four experiments is shown. *p*-value was determined by Student's *t*-test.We analysed as in **A** the effect of E_2_ treatment on interactions between ERα/PI3K and ERα/Src using MCF-7 cells incubated with vehicle (panels a,b) or with E_2_ 10^−8^ M for 5 min (panels c,d) and for 15 min (panels e,f).Quantification of the number of signals per cell was performed as described. The mean ± SEM of four experiments is shown. *p*-value was determined by Student's *t*-test.MCF-7 cells transfected with control siRNA duplexes or with specific ERα siRNA duplexes were controlled for ERα expression by Western blot.Then, ERα/PI3K and ERα/Src interactions were analysed by PLA. The nuclei were counterstained with DAPI (blue).Quantification of the number of signals was performed as described above. The mean ± SEM of four experiments is shown. *p*-value was determined by Student's *t*-test.

Castoria et al. reported that oestrogen rapidly triggers the interaction of ERα with Src as well as PI3K in MCF-7 cells and forming a complex involved in oestrogen non-genomic-induced cell proliferation (Castoria et al, 2001). This result has largely been confirmed by others in several breast cell lines (Cabodi et al, 2004; Fernando & Wimalasena, 2004) as well as in other tissues (Hisamoto et al, 2001). However, all of these results were obtained by immunoprecipitation in cell lines that did not allow the visualization of interactions between proteins. Therefore, the physiological relevance of this signalling pathway remains questionable. To date, immunofluorescence analysis of the complex has been impeded by the fact that only a small population of ERα interacts with Src and PI3K. To circumvent this problem, we used a newly developed technique, PLA. Using PLA, protein–protein interactions can be sensitively and specifically identified using pairs of proximity probes and detected by *in situ* circular amplification, with each red dot representing an interaction (Soderberg et al, 2006). We investigated the ERα/PI3K interaction in the human breast tumour cell line MCF-7 using a rabbit anti-ERα together with a mouse anti-p85 antibody. The ERα/Src interaction was detected using the same anti-ERα together with a mouse anti-Src antibody. Figure 1A shows that ERα interacted with PI3K and Src in the cytoplasm of MCF-7 cells as indicated by the presence of red dots for both antibody pairs (panels a,b). No dots were detected using only one antibody (panels c–e) as confirmed by counting dots per 100 cells (Fig 1B, around 50 dots/cell *vs.* <5). Importantly, the number of red dots increased after 5 min of oestrogenic treatment, then decreased after 15 min. This confirmed that upon oestrogenic treatment, the formation of this complex is rapid and transitory (Fig 1C: compare panels a,b to panels c,d and e,f and Fig 1D). As expected, we observed a decrease in the interaction between ERα/PI3K and ERα/Src in MCF-7 cells upon tamoxifen treatment (Supporting Information Fig S1A and S1B) and ERα knockdown (Fig 1E–G), validating the specificity of the above results. In addition, we performed a set of controls to further validate the specificity of the PLA technology. We tested the interactions between ERα with two known ERα nuclear co-activators, SRC3 and p300 (Acevedo & Kraus, 2003). They were detected exclusively in the nucleus of MCF-7 cells as expected (Supporting Information Fig S2). We previously identified that FAK is also recruited into the complex (Le Romancer et al, 2008) as confirmed by others (Sanchez et al, 2010). Therefore, we studied the interaction of FAK with ERα by PLA. As seen in Supporting Information Fig S3, although FAK interacts with Src, we did not detect any red dots indicating an ERα/FAK interaction. This result is concordant with our previous data showing that the recruitment of FAK into the complex is mediated by its interaction with Src.

**Figure 2:**
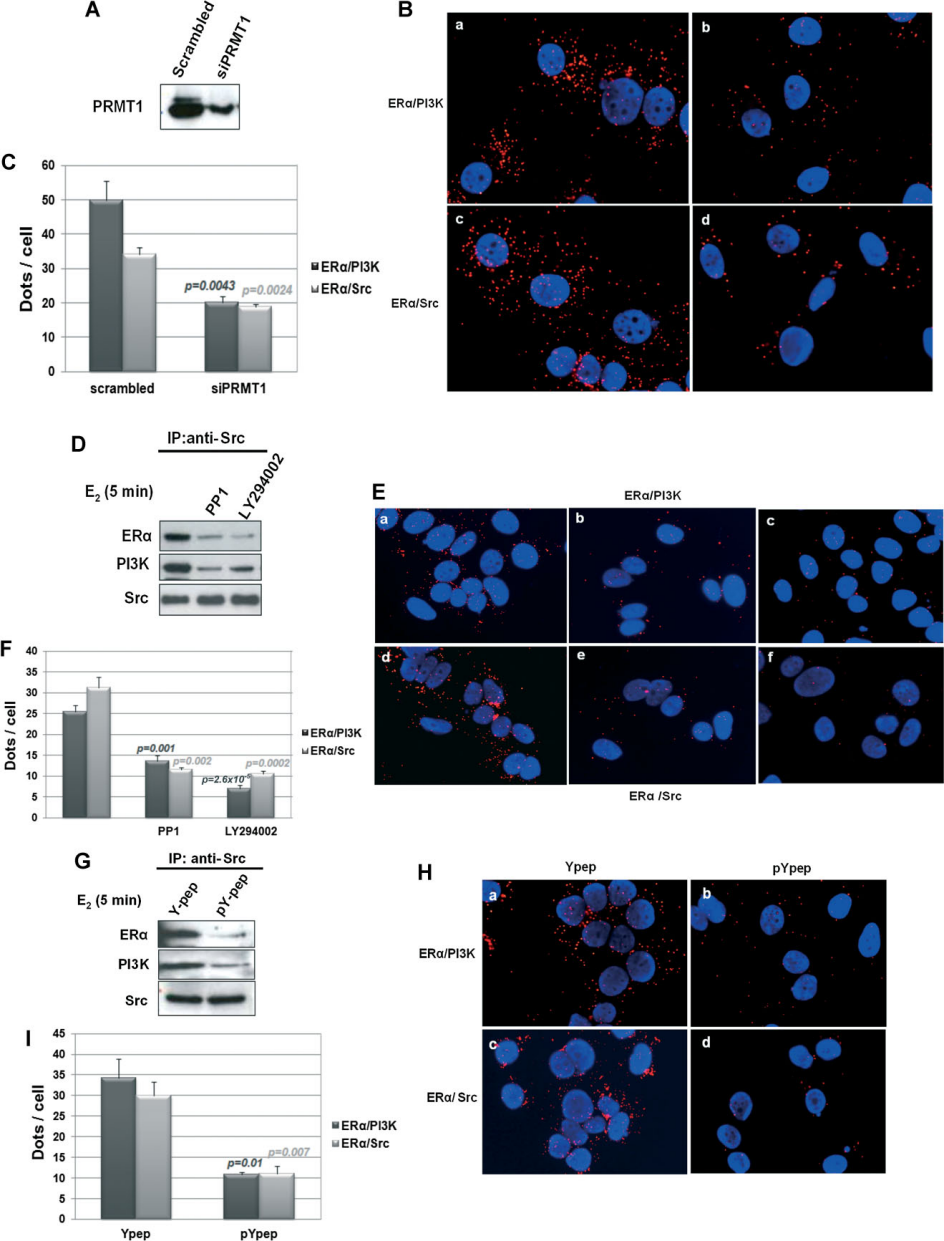
Regulation of ERα/PI3K and ERα/Src interactions in MCF-7 cells detected by PLA MCF-7 cells transfected with control siRNA duplexes or with specific PRMT1 siRNA duplexes were controlled for PRMT1 expression by Western blot.We analysed by PLA ERα/PI3K (panels a,b) and ERα/Src dimers (panels c,d).Quantification of the number of signals was performed as described. The mean ± SEM of four experiments is shown. *p*-value was determined by Student's *t*-test.MCF-7 cells, treated or not with PP1 (5 µM) or LY294002 (20 µM) 15 min before E_2_ treatment, were incubated with the vehicle or with E_2_ for 5 min. Cell lysates were immunoprecipitated with anti-Src and blotted with indicated antibodies.ERα/PI3K (panels a–c) and ERα/Src (panels d–f) interactions were analysed by PLA in cells treated as described above.Quantification of the number of signals was performed for each couple as described. The mean ± SEM of four experiments is shown. *p*-value was determined by Student's *t*-test.MCF-7 cells were incubated with 1 nM of a peptide mimicking hERα 536–541 containing Y537 (Y-pep) or the corresponding phosphorylated peptide (pY-pep) 30 min before E_2_ treatment. Then, cell lysates were immunoprecipitated with anti-Src and blotted with indicated antibodies.From the same experiment, ERα/PI3K (panels a,b) and ERα/Src interactions (panels c,d) were analysed by PLA.Quantification of the number of signals was performed as described. The mean ± SEM of four experiments is shown. *p*-value was determined by Student's *t*-test.

We previously showed that the formation of the ERα/PI3K/Src complex requires the methylation of ERα as well as the kinase activity of Src and PI3K (Le Romancer et al, 2008). Therefore, we performed PLA analysis either using PRMT1 knockdown cells or after the addition of PP1 (Src inhibitor) or LY294002 (PI3K inhibitor). PLA analysis confirmed these results with a significant decrease of red dots (Fig 2A–F). Furthermore, the group of Aurricchio found that a six-amino acid peptide (pYpep) that mimics the sequence around the phosphotyrosine residue in position 537 of the human ERα disrupts ERα/Src interaction and oestrogen-induced proliferation (Varricchio et al, 2007). Indeed, treatment with the phosphorylated peptide induced a notable disruption of the complex, visualized by both immunoprecipitation (Fig 2G) and PLA analysis (Fig 2H and I).

Finally, we confirmed the interactions between ERα/PI3K and ERα/Src using the ERα-positive cell lines CLB-SAV, ZR75.1 and Cama-1 as well as the ERα-negative cell line MDA-MB-231. Supporting Information Fig S4 shows that both complexes were present in the cytoplasm of CLB-SAV and ZR75.1 cells (panels a–d) but not in Cama-1 cells nor MDA-MB-231 cells (panels e–h). Formation of the complex was concordant with the methylation of ERα as we did not detect any oestrogen-induced methylation in either MDA-MB-231 or Cama-1 cells (Supporting Information Figures S4B–D).

All these *in vitro* data clearly validate the PLA technology as a powerful tool to analyse ERα/PI3K and ERα/Src interactions.

### ERα interacts with PI3K and Src in normal breast samples

**Figure 3:**
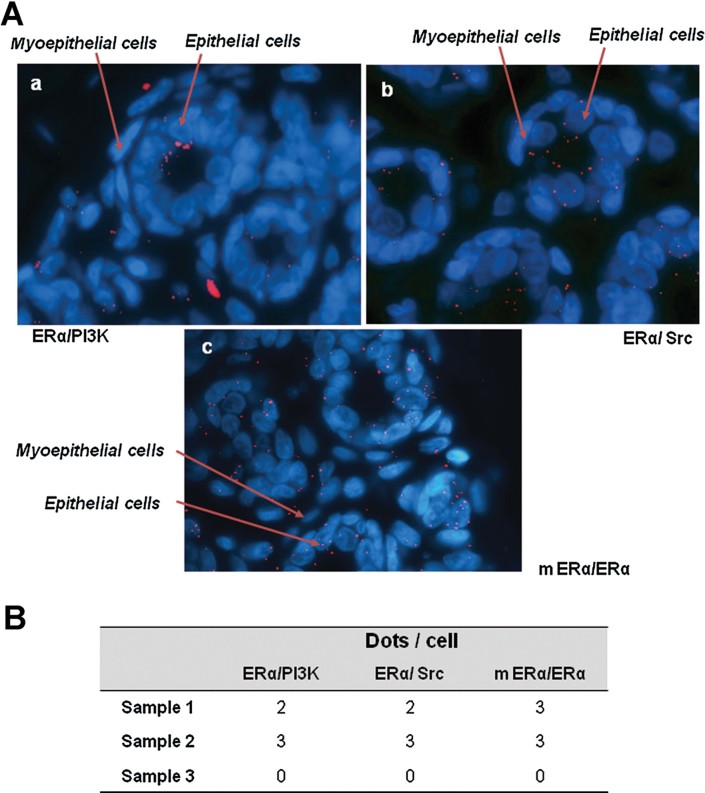
ERα/Src/PI3K complex expression in human normal breast tissue ERα/PI3K, ERα/Src interactions and ERα/mERα were detected with PLA on 3 formalin-fixed human mammoplasty samples. Here is an example of the results obtained on sample 1 for ERα/PI3K interaction (panel a), ERα/Src interaction (panel b) and ERα methylation (mERα/ERα, panel c).Quantification of the number of dots/cell was performed on the three samples for each couple as described.

A crucial question about oestrogen non-genomic signalling concerns its physiological relevance. To approach this issue, we first investigated the presence of the ERα/Src/PI3K complex in three human normal breast samples obtained after mammoplasty. Thus, we performed PLA experiments using the two previously described pairs of antibodies to study the ERα/Src and ERα/PI3K interactions. To correlate these interactions with the presence of methylated ERα, we detected mERα by PLA using rabbit anti-ERα together with the mouse anti-mERα antibody (mERα/ERα). As shown in Fig 3A, we detected ERα/PI3K (panel a), ERα/Src (panel b) and mERα/ERα expression (panel c) in the cytoplasm of epithelial but not myoepithelial cells. The quantification of red dots revealed a low level expression of the complex. This was expected as ERα is faintly expressed in normal breast epithelial cells. We obtained similar results for all three mammary samples (Fig 3B).

### In human breast cancers, the interaction of ERα with both PI3K and Src correlates with ERα methylation and Akt activation

**Figure 4:**
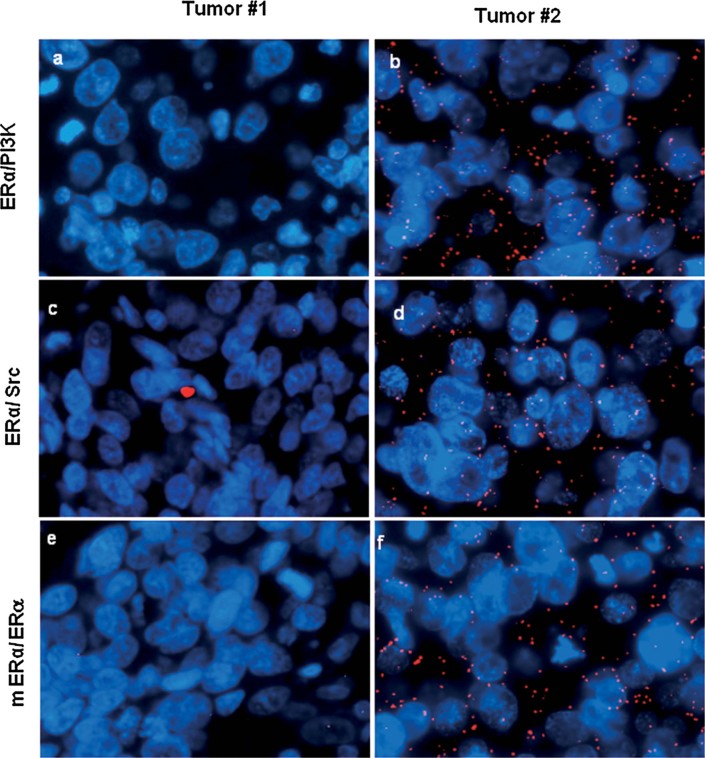
ERα/Src/PI3K complex expression in human tumoral breast samples ERα/PI3K (panels a,b), ERα/Src (panels c,d) and ERα/mERα (panels e,f) expression were detected with PLA on two formalin-fixed paraffin-embedded breast tumours. The nuclei were counterstained with DAPI (blue). The experiments were performed in three serial sections from the same tumour (Obj X63).

We next evaluated the presence of the ERα/PI3K and ERα/Src complexes as well as mERα/ERα expression in invasive breast tumours. The signal for each protein pair varied in intensity from null to a very strong signal. Figure 4 shows two examples of signals we obtained. Tumour #1 did not express the complex whereas Tumour #2 expressed high levels of complex. Of interest, mERα expression correlated with the presence of both ERα/Src and ERα/PI3K complexes as visualized by red dots localized in the cytoplasm of tumour cells.

Subsequently, the study was extended to include 175 invasive breast cancers in 5 tissue microarray blocks (TMA). To perform these highly scaled experiments, we used a different PLA kit, which allows the visualization of brown dots in bright field microscope. We also performed immunohistochemistry analysis using an anti-p-Akt antibody on the same tumour samples in order to confirm that ERα methylation triggers Akt activation. Results from these PLA experiments were quantified by counting at least 400 cells and expressed as the mean number of dots per cell as described in the Material and Methods Section (see Supporting Information Table S1).

**Figure 5:**
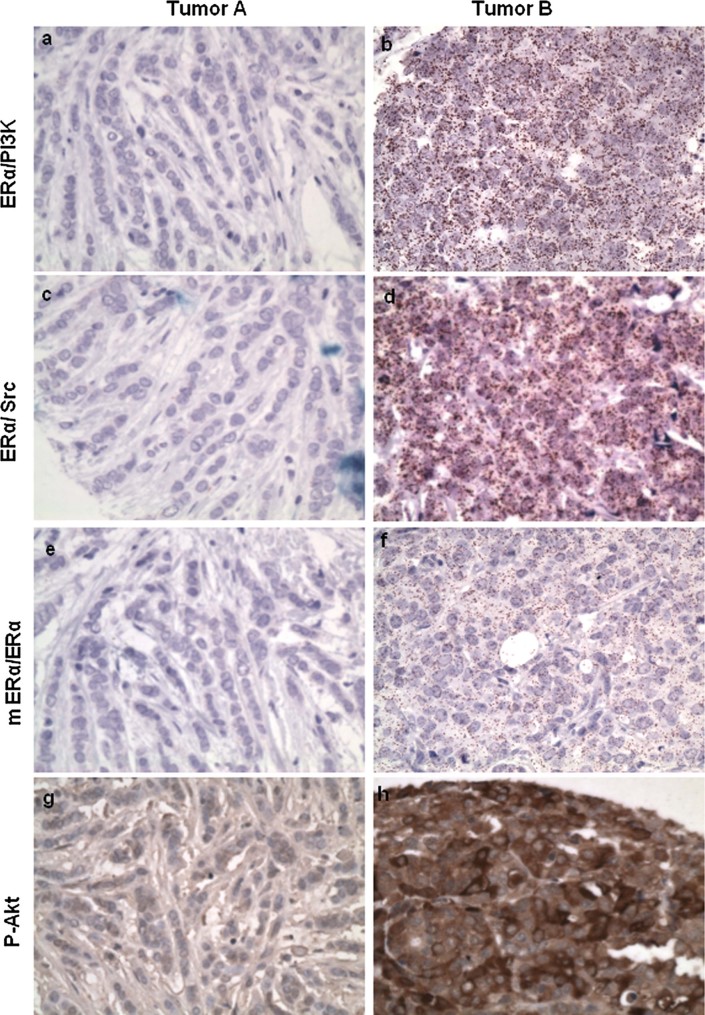
ERα/Src/PI3K complex and p-Akt expression in TMA revealed by bright field PLA For each tumour, we analysed by PLA the levels of ERα/PI3K (panels a,b), ERα/Src complexes (panels c,d) ERα/mERα expression (panels e,f) and P-Akt expression by immunohistochemistry (panels g,h). The experiments were performed in four serial sections from the same tumour.

Interestingly, when we performed a correlation analysis between the different markers, we found significant correlations between ERα/PI3K, ERα/Src interactions and mERα expression (*p* < 0.001; Table 1). This confirms our hypothesis that mERα is responsible for forming the complex. We also discovered statistically significant correlations between each protein pair and P-Akt expression. Figure 5 illustrates representative results showing the high correlation between the expression of this signalling complex and the activation of its downstream effector Akt.

**Table 1:** Correlation analysis between the different markers and p-Akt

ER*α*/PI3K	0.79***	0.75***	0.29***
	ER*α*/Src	0.73***	0.30***
		ER*α*/mER*α*	0.33***
			P-Akt

Correlation studies were performed using the Pearson's coefficient between the couples (ERα/PI3K, ERα/Src and ERα/mERα) and p-Akt.

*** *p* < 0.001.

These data consistently demonstrate that ERα methylation is required for mediating the interaction of the oestrogen receptor with Src and PI3K, which propagates the signal to downstream transduction cascades. Overexpression of mERα and the signalling complex can lead to the hyperactivation of Akt.

### High ERα/Src and ERα/PI3K interactions are associated with clinical factors of poor prognosis

Based on the quantification of dots per cell for each protein couple, we analysed the association between their expression and clinical parameters for 175 breast tumours.

For the expression of ERα/Src, we did not find any association with the status of ERα, PR or HER2. However, age >50 years and menopausal status were significantly associated with a low expression of ERα/Src (respectively 80% and 76% *vs.* 58% and 55% of patients with a high expression of ERα/Src, *p* = 0.003 and *p* = 0.006). ERα/Src expression was also associated with lymph node involvement (42% of patients with a low expression of ERα/Src had lymph node involvement *vs.* 52% of patients with a high expression of ERα/Src, *p* = 0.038; Table 2). Thus, a high expression of ERα/Src was associated with less favourable prognostic factors.

**Table 2:** Distribution of clinical parameters according to groups of ERα/Src expression

	Duolink ERα/Src				Test (*p*)
		
	0–4 (*N* = 79)		>4 (*N* = 96)		
			
	N	%	N	%	
Age at diagnosis (years)					*χ*^2^ (**0.003**)
<50	16	20.3	40	41.7	
≥50	63	79.7	56	58.3	
Menopause					*χ*^2^ (**0.006**)
ND	1		2		
No	19	24.4	42	44.7	
1 Yes	59	75.6	52	55.3	
Tumour size (mm)					*χ*^2^ (0.852)
<20 mm	31	39.2	39	40.6	
≥20 mm	48	60.8	57	59.4	
Histological grade (SBR)					*χ*^2^ (0.505)
1	17	21.5	15	15.6	
2	33	41.8	39	40.6	
3	29	36.7	42	43.8	
Lymph node involvement					Fisher Exact (**0.038**)
N0	34	43.0	42	43.8	
Micro metastasis	12	15.2	4	4.2	
Macro metastasis	33	41.8	50	52.1	
Lympho-vascular invasion					*χ*^2^ (0.119)
Yes	31	39.2	49	51.0	
No	48	60.8	47	49.0	
Oestrogen receptor: % marked cells					*χ*^2^ (0.824)
ND	1		0		
<10%	19	24.4	22	22.9	
≥10%	59	75.6	74	77.1	
Progesterone receptor: % marked cells					*χ*^2^ (0.834)
ND	1		0		
<10%	28	35.9	33	34.4	
≥10%	50	64.1	63	65.6	
HER2 status					*χ*^2^ (0.935)
ND	9		1		
0/+/++FISH−	60	85.7	81	85.3	
++FISH+/+++	10	14.3	14	14.7	

Clinical parameters (age at diagnosis, tumour size, menopausal status, lymph node involvement, SBR grading and hormonal expression) were analysed for the 175 patients included in the TMA study. Association between clinical characteristics and the level of ERα/Src interaction (cut off at 4 dots/cell) was determined using *χ*^2^ test or Fisher's exact test.

Regarding ERα/PI3K expression, we did not find any association with ERα or PR expression. However, a high expression of ERα/PI3K was associated with tumours overexpressing HER2 (25% of tumours with a high expression of ERα/PI3K overexpressed HER2 *vs.* 10% of tumours with a low expression, *p* = 0.019). Moreover, high expression of ERα/PI3K was associated with tumour grade, with more tumours presenting grade 2 or 3 when ERα/PI3K was highly expressed (*p* = 0.014; Table 3).

**Table 3:** Distribution of clinical parameters according to groups of ERα/PI3K expression

	Duolink ERα/PI3K				Test (*p*)
		
	0–7 (*N* = 125)		>7 (*N* = 50)		
			
	*N*	%	*N*	%	
Age at diagnosis (years)					*χ*^2^ (0.720)
<50	39	31.2	17	34.0	
≥50	86	68.8	33	66.0	
Menopause					*χ*^2^ (0.925)
ND	3		0		
No	43	35.2	18	36.0	
Yes	79	64.8	32	64.0	
Tumour size (mm)					*χ*^2^ (0.733)
<20 mm	49	39.2	21	42.0	
≥20 mm	76	60.8	29	58.0	
Histological grade (SBR)					*χ*^2^ (**0.014**)
1	29	23.2	3	6.0	
2	45	36.0	27	54.0	
3	51	40.8	20	40.0	
Lymph node involvement					Fisher exact (0.205)
N0	56	44.8	20	40.0	
Micro metastasis	14	11.2	2	4.0	
Macro metastasis	55	44.0	28	56.0	
Lympho-vascular invasion					*χ*^2^ (0.195)
Yes	61	48.8	19	38.0	
No	64	51.2	31	62.0	
Oestrogen receptor: % marked cells					*χ*^2^ (0.758)
ND	1		0		
<10%	30	24.2	11	22.0	
≥10%	94	75.8	39	78.0	
Progesterone receptor: % marked cells					*χ*^2^ (0.386)
ND	1		0		
<10%	41	33.1	20	40.0	
≥10%	83	66.9	30	60.0	
HER2 status					*χ*^2^ (**0.019**)
ND	9		1		
0/+/++FISH−	104	89.7	37	75.5	
++FISH+/+++	12	10.3	12	24.5	

Clinical parameters (age at diagnosis, tumour size, menopausal status, lymph node involvement, SBR grading and hormonal expression) were analysed for the 175 patients included in the TMA study. Association between clinical characteristics and the level of ERα/PI3K interaction (cut off at 7 dots/cell) was determined using *χ*^2^ test or Fisher's exact test. Significant correlations are highlighted in bold characters.

We found that high expression of mERα/ERα was significantly associated with the youngest people (<50 years old), premenopausal status, higher grade SBR and ERα expression (Supporting Information Table S2).

Altogether, these data strongly suggest that oestrogen non-genomic signalling is associated with common poor prognostic factors for breast cancer patients (Weigel & Dowsett, 2010).

### Survival analysis and predictive value of ERα/Src and ERα/PI3K interactions

**Figure 6:**
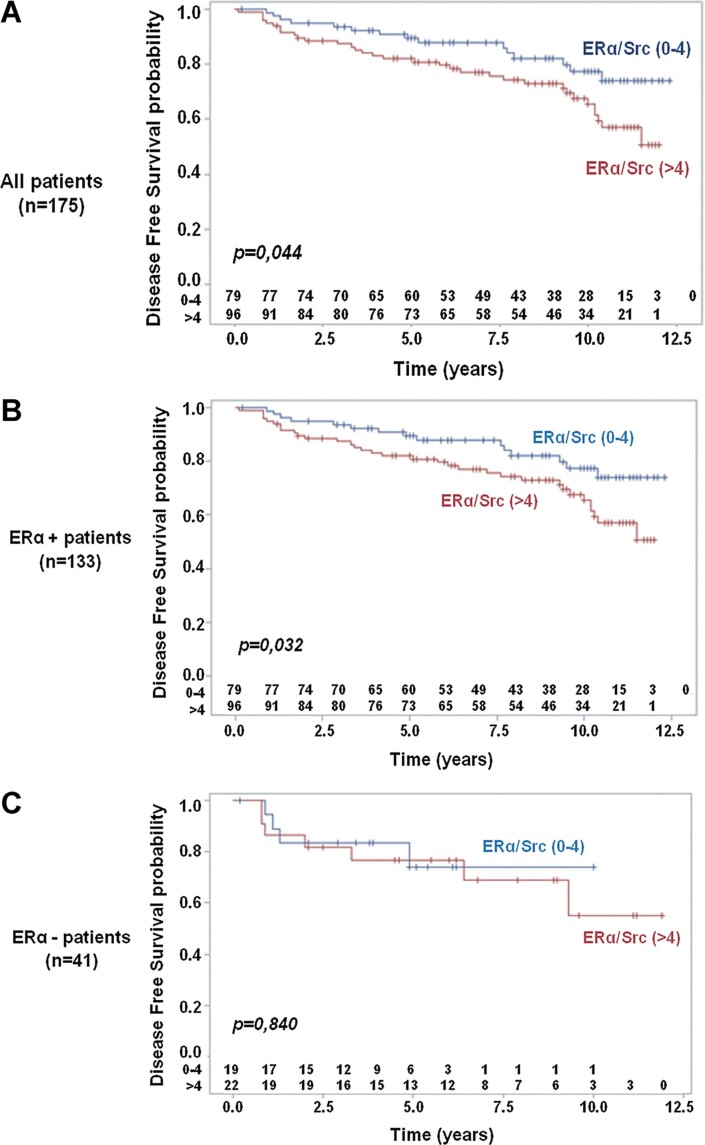
Kaplan–Meier estimates of DFS by ERα/Src expression groups Global population (cut off at four dots per cell).Sub-population of ER-positive cases.Sub-population of ER-negative cases.

We next investigated how ERα/Src and/or ERα/PI3K expression was associated with patient outcomes. Regarding ERα/Src, high expression of this pair was associated with a decreased DFS (Log-Rank test, *p* = 0.044; Fig 6A). Furthermore, within the subgroup of ERα-positive tumours, a high expression of ERα/Src was still associated with a reduced DFS (*p* = 0.032; Fig 6B). For ERα-negative tumours, the number of patients was likely not sufficient to make solid conclusions (Fig 6C). In multivariate analysis, high expression of ERα/Src remained an independent prognostic factor [HR = 1.86, 95% CI (1.01–3.42), *p* = 0.046] adjusted to lymph node involvement [HR = 1.93, 95% CI (1.05–3.56), *p* = 0.035; Table 4]. Of note, parameters like SBR grade, ERα expression and lymph node involvement were not kept as independent prognostic factors in the final model. In terms of overall survival, there was no statistical difference between tumours with high and low expression of ERα/Src (*p* = 0.23; Supporting Information Fig S5).

**Table 4:** Multivariate analysis of DFS integrating ERα/Src expression

Variables	Hazard Ratio	IC95%	*p*-value
Duolink ERα/Src			
0–4	1	–	
>4	1.859	1.01–3.42	0.046
Lymph node involvement			
No	1	–	
Yes	1.929	1.05–3.56	0.035

Hazard ratios for high ERα/Src interaction (score: 0–4) relative to low ERα/Src interaction (score: >4) are shown and for lymph node involvement.

**Figure 7:**
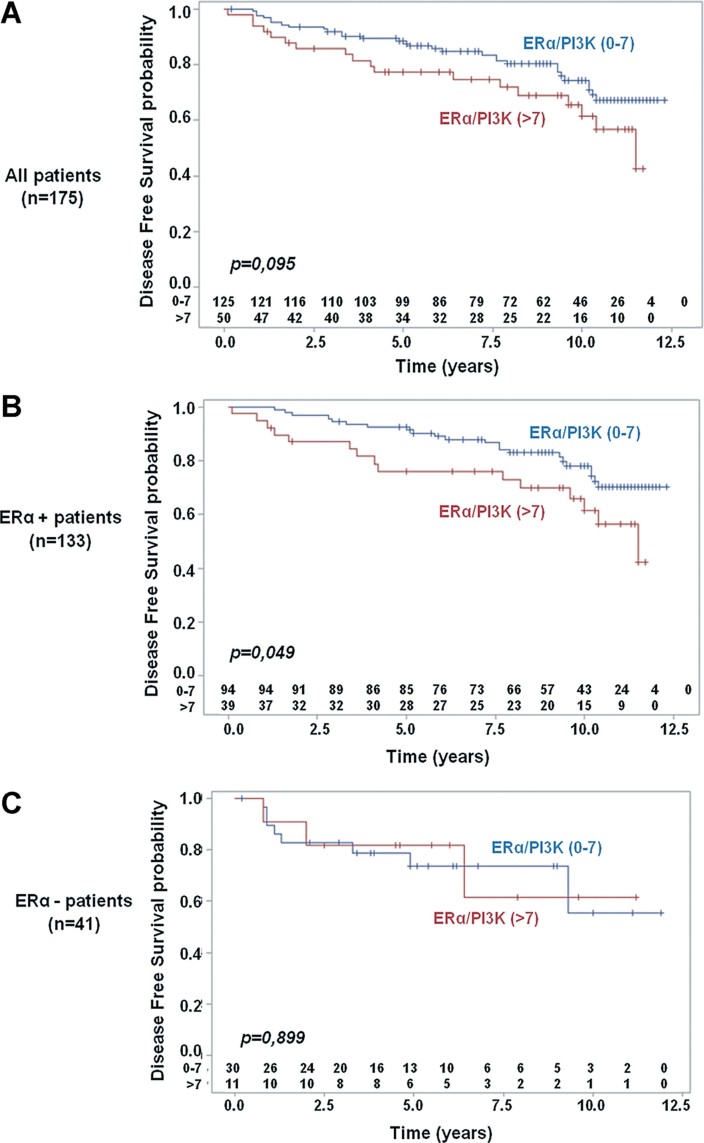
Kaplan–Meier estimates of DFS by ERα/PI3K expression groups Global population (cut off at seven dots per cell).Sub-population of ER-positive cases.Sub-population of ER-negative cases.

We made similar observations for the ERα/PI3K interaction. For all patients, we found no statistical association with either DFS or OS (*p* = 0.096 and *p* = 0.309, respectively), even though a tendency can be observed regarding DFS (Fig 7A and Supporting Information Fig S6). However, for patients with ERα-positive tumours, expression of ERα/PI3K was a prognostic factor for DFS, with a worse prognosis for patients with tumours highly expressing ERα/PI3K, (Log-Rank test, *p* = 0.049; Fig 7B). As for the ERα/Src interaction, the number of patients with ERα-negative tumours was too small to allow solid conclusions (Fig 7C). In multivariate analysis, high expression of ERα/PI3K was found to be linked with DFS [HR = 1.89, 95% CI (1.04–3.42), *p* = 0.037] adjusted to lymph node involvement [HR = 2.07, 95% CI (1.15–3.72), *p* = 0.015; Table 5].

**Table 5:** Multivariate analysis of DFS integrating ERα/PI3K expression

Variables	Hazard ratio	IC95%	*p*-value
Duolink ERα/PI3K			
0–7	1	–	
>7	1.885	1.04–3.42	0.037
Lympho-vascular invasion			
No	1	–	
Yes	2.068	1.15–3.72	0.015

Hazard ratios for high ERα/PI3K interaction (score: 0–7) relative to low ERα/PI3K interaction (score: >7) are shown and for lymph node involvement.

We did not find an association between ERα methylation and patient outcomes as measured by the pair mERα/ERα (Supporting Information Fig S7).

## DISCUSSION

Our results have enabled us to reach several relevant conclusions. Firstly, we have formally demonstrated the presence of the signalling complex described to mediate the rapid action of oestrogen in breast cancer within human breast tissues. This rules out any controversy surrounding the physiological evidence of oestrogen extranuclear action (Warner & Gustafsson, 2006). In fact, we have been able to directly show the association of ERα with its complex partners PI3K and Src in the cytoplasm of mammary epithelial cells. Signals were faint in normal samples and strong in some breast tumour cancers. Furthermore, we report that the presence of methylated ERα statistically correlates with the capacity of ERα to interact with both its partners, PI3K and Src. The precise quantification of these *in situ* interactions has been possible using PLA technology. This allowed the detection and quantification of protein–protein interactions by counting the discrete spot-like signals, each representing one interaction. Since the first step of rapid oestrogen signalling is the formation of a complex containing ERα/Src/PI3K, we evaluated ERα/PI3K and ERα/Src interactions *in situ.* We validated the specificity of this approach by setting up experimental conditions in MCF-7 cells and confirmed by PLA the *in vitro* data on the formation and regulation of the complex ERα/Src/PI3K (Figs 1 and 2, Supporting Information Figs S1–S4). Indeed, we confirmed that oestrogen treatment triggers the rapid and transient interaction of ERα with PI3K and Src. Both interactions increased significantly after 5 min, then decreased after 15 min of E_2_ treatment (Fig 1C and D).

Subsequently, we analysed the expression of the complex in several human breast cancer cell lines and demonstrated a concordance between the levels of ERα methylation and both ERα/PI3K and ERα/Src interactions (Supporting Information Fig S4). This confirmed the data we obtained in MCF-7 cells demonstrating that receptor methylation is a prerequisite for the formation of the complex.

We have used PLA technology to confirm the presence of the ERα/PI3K/Src complex in the cytoplasm of tumoral cells in a cohort of 175 invasive breast cancers. The precise quantification of the signals obtained for each protein pair (ERα/Src, ERα/PI3K and ERα/mERα) clearly shows that formation of the complex was highly increased in a subset of breast cancers. For example, we measured 0–3 dots per cell for the ERα/Src interaction in normal cells, compared to up to 21 dots per cell in tumour samples. We made the same observations for the other two protein pairs (compare Fig 3B with Supporting Information Table S1). The analysis of ERα/Src interaction in 175 breast tumour samples showed that 55% of breast tumours highly express this protein pair (Table 2). Our result is different from those of Welsh et al (Welsh et al, 2012). They analysed ERα cytoplasmic expression by quantitative immunofluorescence on 3200 tumour samples and found that only 1.5% of tumours express cytoplasmic ERα. This discrepancy is probably due to a lack of sensitivity and highlights the use of the PLA technology as a powerful tool to measure oestrogen non-genomic signalling.

Moreover, our work supports the concept that in breast tumours, nuclear and non-nuclear oestrogen signalling can act independently. In fact, we found the ERα/Src/PI3K complex in tumours negative for nuclear ERα. In our cohort of 175 patients, 22 (54%) of the 41 ERα-negative tumours expressed a high level of ERα/Src (Table 2). This is in agreement with our previous analysis of ERα methylation in a different cohort of 164 breast tumours where we found that 53% of ERα-negative tumours expressed hypermethylated ERα (Le Romancer et al, 2008). Kumar et al also found ERα expression in the cytoplasm of tumours classified as ERα-negative. They explained this unusual localization by the sequestration of ERα into the cytoplasm through a natural variant of MTA1, MTA1s (metastasis-associated antigen 1 short form; Kumar et al, 2002). However, they did not demonstrate that a cytoplasmic pathway was activated in these tumours. We have confirmed the association of ERα methylation and its interaction with PI3K and Src in line with our previous results obtained in cellular models. This suggests that ERα methylation is a key step for ERα/Src/PI3K complex formation. Interestingly, the formation of the complex correlates with the activation of Akt as measured by the status of Akt phosphorylation. This strongly indicates that in breast tissues, oestrogens activate cytoplasmic phosphorylation cascades by triggering the methylation of ERα and the recruitment of Src and PI3K. This finding introduces a new concept: the rapid oestrogen pathway is operative *in vivo* and deregulated in a subset of breast cancers.

Our second important result shows that the association level of ERα with both Src and PI3K correlates with other prognostic factors, such as high SBR grade and lymph node involvement. Thereby, this strongly suggests that activation of the cytoplasmic signalling pathway could constitute a marker of tumour aggressiveness. The association between the ERα/Src interaction and lymph node metastasis is particularly interesting and could involve FAK activity as we have demonstrated that FAK is also recruited with ERα/Src/PI3K upon oestrogenic stimulation (Le Romancer et al, 2008).

Due to the major role that ERα plays in the development and progression of breast cancer, the oestrogen signalling pathway has been studied in depth. Current endocrine therapies for breast cancer are mainly based on targeting the ERα signalling pathway: reducing oestrogen abundance with aromatase inhibitor (Baum et al, 2002; Johnston & Dowsett, 2003), antagonizing ERα function with tamoxifen and raloxifene (Jensen & Jordan, 2003) or down-regulating ERα expression with fulvestrant (MacGregor & Jordan, 1998). However, resistance to endocrine therapies is one of the major barriers to the successful treatment of breast cancer (Musgrove & Sutherland, 2009; Yamashita, 2008). There is a real need to find markers predicting resistance to treatment. Currently, ERα expression in the nucleus is the only known biomarker of response to endocrine therapy. As a consequence, non-genomic ERα signalling has never been assessed in clinical practice.

Aberrant activation of the PI3K/Akt/mTOR pathway has been found in many types of cancer and thus plays a role in breast cancer proliferation and anti-cancer resistance (Ghayad & Cohen, 2010). It is clear that activation of this signalling pathway triggers a cascade of biological events such as cell growth, proliferation, survival and migration, which contribute to tumour progression. Therefore, this pathway is an attractive target for the development of anti-cancer molecules and several kinase inhibitors have already been developed. Several of these inhibitors are currently under clinical evaluation (Ghayad & Cohen, 2010). The tyrosine kinase Src has also been considered as a potential target and Src inhibitors like dasatinib or bosutinib have been tested in phase II clinical trials (Araujo & Logothetis, 2010). However, so far, the effects have been quite disappointing. In fact, dasatinib used as a single agent has limited activity in patients with triple-negative breast cancer (TNBC; Finn et al, 2011) or patients with heavily treated metastatic breast cancer (Campone et al, 2012) and it advances ERα-positive tumours. However, *in vitro* studies show that combining anti-oestrogen and Src inhibitor enhances growth inhibition (Chen et al, 2011). Moreover, clinical trials are ongoing to combine dasatinib with other therapies (Mayer & Krop, 2010). However, even if clinical studies give satisfactory results, there remains a real need to identify biomarkers that will predict which patients could benefit from these inhibitors either alone or in combination.

We can speculate that the deregulation of oestrogen non-genomic signalling may open up new perspectives for anti-cancer treatment strategy. However, our patient population was small and usual prognostic factors (such as tumour size) were not found to be significant in the current retrospective analysis. Independent validation is required and this can be done in the context of randomized clinical trials with endocrine therapy where the oestrogen non-genomic signalling can be assessed retrospectively. Furthermore, our work suggests that the non-genomic signalling pathway may be taken into account to optimize targeted therapies. In the metastatic setting, as described above, PI3K and/or Src appear to be promising targets for treatment. We can thus imagine targeting the entire ERα/Src/PI3K complexes. The disruption of the complex containing mERα/Src/PI3K has already been shown to decrease cell proliferation. Consistent with this hypothesis, the work by Aurricchio et al has shown that disrupting the ERα/Src interaction with a peptide impairs complex formation and the proliferation of tumour cells both *in vitro* and in xenografted mice (Varricchio et al, 2007). We speculate that combining endocrine therapies with Src inhibitors and/or PI3K inhibitors based on the level of ERα/Src or ERα/PI3K interactions may be clinically relevant. This concept has to be validated in large prospective clinical studies.

For TNBC, which account for approximately 15% of all breast cancers (Foulkes et al, 2010) and for which specific targets are lacking, determining which pathways are activated is important. When we analysed the DFS of patients with ERα-negative tumours according to ERα/Src intensity staining in a Duolink experiment (0 *vs.* 1–2), we identified a subpopulation of patients that did not display oestrogen non-genomic signalling and who did not relapse (Supporting Information Fig S8). However, few patients do not express ERα/Src (*n* = 10 *vs. n* = 31 patients with intensity >0) and the difference is not statistically significant (*p* = 0.074). For patients with tumours expressing ERα/Src, we assume that this complex may become a new target for treatment. Again, this assumption has to be validated in a prospective clinical trial.

In summary, this work is proof of concept that the oestrogen non-genomic pathway, represented by the formation of the ERα/Src/PI3K protein complex, potentially constitutes a novel tumour biomarker to predict survival and/or response to targeted agents. These encouraging results raise the interest for further clinical studies with large patient populations. We thus planed to test the effects of combining endocrine therapy plus kinase inhibitors such as PI3K/mTor inhibitors or Src inhibitors in preclinical studies as well as in clinical trials.

## MATERIALS AND METHODS

### Cell Culture and Transfections

MCF-7, CLB-SAV, MDA-MB-231 and ZR75-1 cells were maintained at 37°C in Dulbecco's Modified Eagle's Medium (DMEM) supplemented with 10% fetal calf serum and 1% non-essential amino acids. Cama-1 cells were maintained at 37°C in RPMI supplemented with 10% serum. CLB-SAV is an epithelial cell line established by C. Caux (Centre Leon Berard, Lyon, France) from ascitic fluid of a 58-year old patient with lobular breast carcinoma. We have already shown that this cell line expresses ERα and mERα (Le Romancer et al, 2008). ZR-75 and Cama-1 express ERα although MDA-MB-231 does not express the receptor (Supporting Information Fig S4C).

To study the effect of hormones on ERα methylation, the cells were treated for different times with E_2_ or tamoxifen (Sigma). When stated, MCF-7 cells were treated with Src inhibitors PP1 or with PI3K inhibitor LY 294002 (Calbiochem).

The siRNA sequence targeting PRMT1 has already been described (Le Romancer et al, 2008). The siRNA sequences targeting ERα correspond to the coding regions 854–872 (siERα1) and 1137–1154 (siERα2) and have been mixed before transfection.

### Antibodies

Antibodies are listed in Table 6A and B.

**Table 6:** List of used antibodies

A: Antibodies for PLA analysis			

Antibodies	References	Species	Dilution
ERα (1D5)	7047 (Dako)	Mouse	1/50
ERα (HC20)	Sc-542 (Santa Cruz)	Rabbit	1/75
PI3K p85	Ab22653 (Abcam)	Mouse	1/30
Src (B12)	Sc-8056 (Santa Cruz)	Mouse	1/150
mERα	Home made	Mouse	1/50
p300 (NM11)	Sc-32244 (Santa Cruz)	Mouse	1/100
SRC3	Sc-13066 (Santa Cruz)	Rabbit	1/50
FAK (A17)	Sc-557 (Santa Cruz)	Souris	1/50

B: Antibodies for immunoprecipitation and Western blotting				

Antibodies	References	Species	Dilution Western Blotting	IP
mERα	Home made	Mouse		1 µg
ERα (60C)	#04-820 (Millipore)	Rabbit	1/1000	
PRMT1	#24-333 (Upstate)	Rabbit	1/4000	
PI3K p85	#06-195 (Upstate)	Rabbit	1/9000	
c-Src (B12)	Sc-8056 (Santa Cruz)	Mouse		1 µg
Src (36D10)	#2109 (Cell signaling)	Rabbit	1/1000	

### Immunoprecipitation and Western blotting

After treatment, cells were lysed using RIPA buffer (50 mM Tris–HCl, pH 8, 150 mM NaCl, 1 mM EDTA, 1% NP-40, 0.25% deoxycholate) supplemented with protease inhibitor tablets (Roche Molecular Biochemicals) and phosphatase inhibitors (1 mM NaF, 1 mM Na_3_VO_4_, 1 mM β-glycerophosphate). Protein extracts were incubated with primary antibodies overnight at 4°C with shaking. Protein A-agarose or Protein L-agarose beads were added and the mixture was incubated 2 h at 4°C then washed three times with lysis buffer. After separation on SDS–PAGE, proteins were analysed by Western blotting.

### Human breast cancer samples collection

The tumours from 175 CLB patients with invasive non-metastatic breast cancer whose clinical and biological data were available from the regularly updated institutional database were analysed. Written informed consent was obtained from each patient and the study protocol was approved by the institutional ethics committee. Patients's characteristics are presented in Supporting Information Table S3. Median age was 55 (range 27–87) and the majority of patients were post-menopausal (65%). In our study, tumours exhibiting less than 10% of ER positive cells qualify for ER negative tumours. The patient follow up was performed as routine practice.

### Immunohistochemistry

Paraffin embedded tumours tissue fixed in Formalin were used for analysis. The pathologist selected representative areas from breast invasive carcinomas. Triplicates from each tumour were inserted in a TMA block which contained 40 tumours. Five TMA (200 tumours) were analysed. The blocks containing invasive carcinoma were serially sectioned at a thickness of 4 µm. After deparaffinization and rehydratation, tissue sections were boiled in 10 mM citrate buffer ph6 using a water bath at 97°C for 40 min.

For blocking endogenous peroxidases, the slides were incubated in 5% hydrogen peroxide in sterile water. The slides were then incubated at room temperature for 1 h with the anti-p-Akt monoclonal rabbit antibody (Ref: 2118.1, Epitomics, Burlingame, California) diluted at 1/50. After rinsing in Phosphate Buffer Saline, the slides were incubated with a biotinylated secondary antibody bound to a streptavidin peroxidase conjugate (Envision Flex kit Ref: K800021-2, Dako, Trappes, France). Bound antibody was revealed by adding the substrate 3,3-diamino benzidine. Sections were counterstained with hematoxylin.

### Proximity ligation assay

This innovent technology developed by Olink Bioscience (Sweden) allows visualizing protein/protein interactions *in situ* and has been firstly published in 2006 (Soderberg et al, 2006).

#### Fluorescence revelation

MCF-7 cells (9.5 × 10^5^), CLB-SAV cells (20 × 10^5^), MDA-MB-231 cells (10 × 10^5^), Cama-1 cells (10 × 10^5^) and ZR75-1 cells (10 × 10^5^) were grown on coverslips into 12-well plates. Cells were fixed in methanol for 2 min, washed twice in PBS. For tumours analysis, blocks containing breast tumours of 4 µm thick sections were cut, deparaffinized and subjected to antigen retrieval by water bath in citrate buffer (pH 6.0) during 40 min, and at rest during 20 min. Then, the slides were treated according to the manufacturer's instructions (Duolink II Fluorescence, Olink Bioscience, Sweden). Firstly, the samples were saturated using the blocking solution, then different couples of primary antibodies (rabbit and mouse in our case) were incubated 1 h at 37°C. After two washes in PBS, the PLA minus and plus probes (containing the secondary antibodies conjugated with complementary oligonucleotides) were added and incubated 1 h at 37°C. The next step allows the ligation of oligonucleotides if the two proteins are in close proximity thanks to the ligase during an incubation of 30 min at 37°C. Then, after two washes, the addition of nucleotides and polymerase, allows amplification by rolling-circle amplification (RCA) reaction using the ligated circle as a template during an incubation of 100 min at 37°C. The amplification solution also contains fluorescently labelled oligonucleotides that hybridize to the RCA product. Then, the samples were let drying at room temperature in the dark and were mounted with Duolink II Mounting Medium containing Dapi, then analysed on fluorescence microscope.

#### Bright field revelation

For TMA analysis, we used another revelation kit (Duolink II Brightfield) that allows detecting the signal by colorimetry under visible light. The first step is to avoid peroxidase quenching incubating the samples 5 min at room temperature, with a hydrogene peroxide solution. The following steps are identical to what was described before. For the detection, the probes are labelled with horseradish peroxidase after two washes in high purity water; nuclear staining solution is added on slides and incubated 2 min at room temperature. After washing the slides 10 min on running tap water, the samples were dehydrated in ethanol, then in Xylene solution. Samples were mounted in non-aqueous mounting medium and then analysed with a Bright field microscope.

### Image acquisition and analysis

The hybridized fluorescent slides were viewed under a Leica DM6000B microscope. Images were acquired under identical conditions at objective X63. On each samples, 100 cells were counted. Analyses and quantifications of these samples were performed using Image J software (free access). This software allows counting dots on 8 bits image. The plugin “Counter cells” allows analyzing cells number.

The hybridized Bright field slides were viewed under a Leica DMLB microscope. Images on three independent zones of each tumour on TMA were acquired under identical conditions at objective X40. On each tumour, 500 tumourous cells were counted. We have done the analysis on TMAs blocks which include three cores of 600 µm for each tumour. In fact, each core is obtained in a different area of the tumour so that we try to take in account tumour heterogeneity. For PLA assessment, we make an average of the staining while counting the three cores. We do not choose focal areas on the cores. In the vast majority of our cases, a homogenous staining within the three cores was observed except for ten tumours. In these cases, the average of staining obtained from the three cores was used.

Thereafter, analyses and quantifications of these samples were performed using Duolink ImageTool software (developed by Olink Bioscience)

### Statistical analysis

#### Correlation analysis

Correlations between the three biomarkers were studied. Furthermore, correlations between each couple (ERα/Src, ERα/PI3K, ERα/mERα expression) and p-Akt were also performed. The software produces a graph which represents the correlation between each variable 2 by 2. The Pearson's correlation coefficient were presented and the stars identify its significance threshold (*p < 0.05; **p < 0.01; ***p < 0.001).

#### Descriptive analysis

Thanks to concertation between clinicians and biologists, thresholds were defined to distinguish in an optimal way high and low expression of ERα/Src (≤4/>4 dots per cells), ERα/PI3K (≤7/>7) and ERα/mERα (≤3/>3). Distribution of clinical parameters (cancer subtype, clinical, histological and immunohistochemical data) was compared between ERα/Src, ERα/PI3K and ERα/mERα expression groups, using Pearson's *χ*^2^ test or Fisher's exact test. Same comparisons were made according to intensity levels but only results about dots per cells are presented in this article.

#### Survival analysis

Overall survival defined as time from diagnosis to death or date of last follow-up and DFS defined as time from diagnosis to death or relapse or date of last follow-up (for censored patients) were studied.

Survival distributions were estimated by Kaplan–Meier method and compared between expression's level groups using the Log-Rank test.

To evaluate a possible relationship between DFS and ERα/Src expression, univariate Cox proportional hazard regression models were built by considering ERα/Src expression and some covariates, approved to be prognostic of DFS (tumour size, lymph node status, RE, RP, HER2 status and SBR grade). All interactions between variables (significant at 20%) were tested and only significant ones (*p* ≤ 0.05) were entered in the initial multivariate Cox model in addition to variables statistically significant in univariate analysis at 20% level. A backward manual selection procedure was used to lead to the final model by removing non-significant variables (*p* > 5%). Similar DFS study was achieved with ERα/PI3K marker.

**The Paper explained**

PROBLEM:

At breast cancer diagnosis, only patients expressing ERα in the nucleus (classified ERα-positive) are treated by endocrine therapy and unfortunately, some patients die after developing resistance to the treatments. However, a few papers have shown that ERα could be present in the cytoplasm of breast tumours. Furthermore, in cellular models, it has been well established that oestrogen activates extranuclear pathways through the recruitment of ERα/Src/PI3K and activation of downstream kinases as Akt. Our lab has shown that arginine methylation of ERα is a prerequisite for non-genomic signalling triggering association of ERα with Src and PI3K in breast cancer cells. The aim of this work was to assess whether oestrogen non-genomic signalling occurs in human breast tumours and if this signalling pathway could constitute a new therapeutic target.

RESULTS:

To approach oestrogen non-genomic signalling *in vivo*, we studied ERα interaction with Src and PI3K using the technology of PLA that allows detecting protein–protein interactions *in situ*. First, we were able to recapitulate the data about the complex formation containing ERα/Src/PI3K that were obtained with other techniques. We then investigated oestrogen non-genomic signalling in breast tumours. We found that ERα interacts weakly with Src and PI3K in human normal epithelial cells and strongly in some tumour samples. These interactions are independent of the presence of ERα in the nucleus but correlate with the level of methylated ERα. The analysis of 175 tumours showed that ERα/Src, ERα/PI3K and mERα expression is correlated with Akt activation suggesting strongly that this pathway is operative *in vivo*. Interestingly, we found that the high expression of ERα/Src and ERα/PI3K is an independent poor prognostic marker associated with reduced disease free survival

IMPACT:

This is the first evidence that oestrogen non-genomic signalling occurs in breast tissue and is deregulated in a subset of breast tumours. The analysis of ERα/Src and ERα/PI3K interactions could be a new relevant tool for pathologist at diagnosis. We hypothesize that, based on the level of ERα/Src and ERα/PI3K interactions, the clinician could orientate the treatment towards Src or PI3K inhibitor associated with classical treatment for breast tumours.

All statistical analysis were performed using SAS® software, v 9.3 (SAS institute Inc, Cary, NC, USA) except the correlation study, carried out with R software.

## Supplementary material

Detailed facts of importance to specialist readers are published as ”Supporting Information”. Such documents are peer-reviewed, but not copy-edited or typeset. They are made available as submitted by the authors.
