# Supplementary material for: Activation of rapid oestrogen signalling in aggressive human breast cancers
Source: EMBO Mol Med. 2012 Oct 15;4(11):1200–13. doi: 10.1002/emmm.201201615 (PMC3494876; doi:10.1002/emmm.201201615)
Supplement: Supplementary file 1 [file emmm0004-1200-SD1.pdf]

EMBO
Molecular Medicine

Manuscript EMM-2012-01615

Activation of rapid estrogen signaling in aggressive human breast cancers

Coralie Poulard, Isabelle Treilleux, Emilie Lavergne, Katia Bouchekioua-Bouzaghou, Sophie Goddard-Léon, Sylvie Chabaud, Olivier Trédan, Laura Corbo and Muriel Le Romancer

Corresponding author: Muriel Le Romancer, Centre de recherche en Cancérologie de Lyon, UMR INSERM 1052, CNRS5286

Review timeline:

Submission date:	12 August 2011
Editorial Decision:	09 September 2011
Resubmission:	31 May 2012
Editorial Decision:	22 June 2012
Revision received:	14 August 2012
Editorial Decision:	28 August 2012
Revision received:	04 September 2012
Accepted:	05 September 2012

Transaction Report:

(Note: With the exception of the correction of typographical or spelling errors that could be a source of ambiguity, letters and reports are not edited. The original formatting of letters and referee reports may not be reflected in this compilation.)

1st Editorial Decision 09 September 2011

Thank you for the submission of your manuscript "Estrogen non genomic signaling occurs in breast tissue and is deregulated in breast tumors". We have now heard back from the three referees whom we asked to evaluate your manuscript.

As you will see from the enclosed reports, the referees acknowledge the potential interest of the study and reviewer #2 is rather positive about the manuscript. However, reviewers #1 and #3 are much more reserved and have raised significant issues, generally finding the approach promising but too incomplete to be published in EMBO Molecular Medicine. The main criticisms concern the clinical relevance and the conclusiveness of the data, especially the statistical significance of PLA experiments.

Given the too limited support of the reviewers and the fact that EMBO Molecular Medicine can only invite revision of papers that receive enthusiastic support from a majority of referees, I am afraid I see little choice but to return the manuscript with the decision that we cannot offer to publish it.

I am sorry to have to disappoint you on this occasion, and hope that this negative decision does not prevent you from considering EMBO Molecular Medicine for the publication of future studies and that the referee comments are helpful in your continued work in this area.

Yours sincerely,

Editor
EMBO Molecular Medicine

***** Reviewer's comments *****

Referee #1 (Comments on Novelty/Model System):

Better controls need to be included to confirm that PLA doesn't occur when two proteins are expressed in the same cell (i.e in the presence of tamoxifen, or between ER and a non-related growth factor).

Referee #1 (Other Remarks):

This manuscript applies the PLA (proximity ligation assay) to assess the interaction between ER and Src/PI3K in the non-genomic signalling of breast cancers. The authors show that PLA can produce discrete foci representative of amplified products that result from protein proximity. They perform a number of controls and extend their findings to primary material to show that methylated ER and Src/PI3K interact in tumors, including those thought to be ER negative.

Although this is a potentially interesting and relevant study, there are major gaps that need to be filled before this can be published. Also, I am a little unclear of the clinical significance of using PLA over traditional methods, which I explain below.

- The use of PLA for protein-protein interaction is novel and the application to study non-genomic interactions is a very nice way of using this technology. However, I'm still unsure how specific it is. In all cases, a series of dots are revealed which may still be non-specific interactions. The only time the dots are not seen is when one of the protein halves is missing (i.e. in ER negative cells, or after silencing ER). However, is it not possible that any proteins could be shown to be in proximity in a small fraction of the cells in a cell population? The addition of E2 does increase dot formation, but this could simply be due to the sequestration of ER by heat shock protein in untreated conditions. A much better control would be to repeat this experiment in the presence of an antagonist (such as tamoxifen), where ER is still present but is unlikely to interact with growth factors intermediates. In addition, it is essential that a non-interacting growth factor be tested (one that is expressed in the cells). Without this, we may be shown the few cell examples where protein aggregates have formed between numerous proteins, including ER and Src/PI3K.
- I am a little dubious about the data when only shown a couple of cells as examples. Can this data be quantitated across an entire cellular population and plotted graphically? Some of this is provided in Supplementary figures but should be included in the main figures.
- Does the methylated ER recognise total ER? It seems unlikely that methylated ER exists in 66% of ER negative tumors. Is it not more likely that the antibody recognises a methyl group on another protein? Is there a control experiment that can be done to prove that this is specific......i.e. would siRNA to ER reduce the amount of detected methyl-ER?
- I appreciate the desire to establish a new approach for defining ER negative tumors that may respond to treatments. However histological approaches use a very low cut-off (only 1% of cells need to be positive for ER to be called an ER positive tumor). If PLA is more sensitive, which I am happy to believe, then it needs to be shown that the PLA positive (histology negative) tumors or cell line models, respond to antiestrogens. If this doesn't happen then the whole concept is moot. It is well established that cohorts of ER negative tumors gain zero benefit from antiestrogen treatment, so if PLA can identify low level ER, yet the tumors gain no benefit from ER antagonists, I'm unsure of the benefit of using PLA. If the authors can find even a single cell line model that is ER negative by histology, but positive by PLA and responds to antiestrogens, I will be convinced.
- Similarly, the comment about triple negative tumour is redundant. There is no clinical benefit in triple negative tumors to ER antagonists. As such, it is unlikely that PLA will reveal a subpopulation that can gain any clinical benefit. If the authors can convince me that the clinical data and current paradigms are wrong, I would be willing to listen.

Referee #2 (Comments on Novelty/Model System):

For the first time the authors visualise the estradiol-dependent ERalpha/PI3K/Src complex, which has a key role in regulating a proliferative signaling network. This complex is now found not only in hormone-responsive cell lines but also in normal human breast samples,human mammary cancer samples, adipocytes. The role of receptor methylation in regulating the complex assembly is demonstrated.The authors observe a correlation between the level of methylated receptor, but not the nuclear receptor, and the intensity of the complex in breast cancer.
Interestingly, the majority of the analyzed breast tumors classified as ERalpha negative express the cytoplasmic complex implicating that the complex or the methylated receptor could be additional markers of breast cancer progression. The utility of these assays is also supported by the observed increase of axillary lymph node involvement with increased level of arginine methylated receptor.

Referee #2 (Other Remarks):

Use of a new method, the Proximity Ligation Assay, allows to clearly visualize in a variety of conditions and cell types a signaling complex which is specific for the nongenomic proliferative action of estradiol and requires ERalpha arginine methylation.Of remarkable clinical relevance is the presence of the complex in receptor negative human breast cancers and the increased expression of the modified receptor in tumors with increased axillary involvement. The report could be improved if some of the figures in the supplemental material are moved to the main text. This goal might be easily realized if this report will be published as a full research article. Alternatively, at least Fig S1 and S5 should be included in Fig 1B and D, respectively. The spelling of some authors in the text, but not in the references, is wrong.

Referee #3:

Non-genomic signaling by ER and other nuclear receptor family members is well established. M. Le Romancer and colleagues have published previously (Mol. Cell 2008) on a methylated cytoplasmic ERa that they showed in this prior publication to be present in ER-positive breast cancer MCF7 cells, and at a low level (weak level by IHC with their ER arg260 methylation-specific antibody) in normal epithelial breast cells and usually at higher stain intensity by IHC in a significant proportion of the 164 human breast tumors they examined in the 2008 paper. They saw no correlation between methylated cytoplasmic ERa and whether breast tumors were ERa-positive or negative, with 55% of tumors showing methylated ERa and 18% of tumors showing high levels of methylated ERa belonging to ERa-negative cases. Further, they showed that this methylation of ERa was very transient after estrogen treatment of cells, and that it resulted in the formation of a complex of this cytoplasmic methyl-ERa (denoted mERa) with c-Src, the p85 subunit of PI3K, and FAK. This data was based mostly on immunoprecipitation studies and IHC in cells and tumor tissue. Because, the population of the cytoplasmic/membrane ERa is very small, and therefore "only a small population of ERs interacts with Src and PI3K" as stated by the authors on p.5 in the current paper, they use PLA (Proximity ligation assay) to study its presence in cells and interaction of this ERa with PI3K and ERa with Src in situ. This PLA assay has been used by others also to study endogenous protein complexes in situ (ref.16, Soderberg et al. and others). This is a worthwhile approach for documenting in situ interaction of ERa and PI3K, and ERa and Src in the few cell lines examined, but many important aspects are not analyzed sufficiently quantitatively, and the findings only confirm prior published interactions done before by IP. No new aspects are addressed or illuminated. Some clinical samples (n=21) are also studied here by IHC for cytoplasmic ERa.

Major Comments and Questions

1.Throughout the manuscript, the authors need to more quantitatively analyze the data and determine statistical significance. For example, based on data in Table 1, the authors talk about "high expression of methyl-ERa and expression levels of the complexes" (p.9 top), but this needs to be done with rigorous statistical assessments. The authors use a cut-off of less than 1 dot per cell (low) and more than 1 dot per cell (high), but is this most appropriate since dots per cell range from

0 to 10? Is there a gradation for more than 1 dot that could be evaluated? I think a better statistical analysis should be performed to determine is a correlation truly exists and what is its level of significance based on a rigorous analysis of the data.

2.The authors conclude on p. 9 top that "it appeared that the expression of methyl-ERa increased with the axillary lymph node involvement (Table S1)". But Table S1 is based on only 12 patients with lymph node metastasis. This is a small number of patients to be meaningful in drawing any conclusions. Also, more clinical information on the patients and tumors is needed. For example, number of nodes involved, not just positive or negative for nodes. And would be valuable to provide information on stage of the breast tumors and treatment the patients might have been receiving.

3.Why do the authors (p.8 bottom and Table 1) study mERa (methyl-ERa) only in ER-negative breast tumors since mERa is found in a large fraction of ERa-positive breast tumors based on their prior 2008 publication? The authors now show ERa/Src and ERa/PI3K interaction by PLA assay in only 1 ER-positive tumor (Fig.2A) and in 1 ER-negative breast tumor (Fig.2B).

4. What are the relative amounts of nuclear ERa and cytoplasmic mERa in the breast cancer cell lines studied (MCF7, CLB-SAV cells) and in the human mammoplasty samples, and in the human tumor specimens? There is no quantitation of this. I realize that this is not straightforward since different antibodies are used, but I think the authors should try to give some information about this in this manuscript.

5. Interactions by PLA are done only at 1 time point, 5 minutes, as in their 2008 paper. Even though methylation and downstream events are said to be very transient, based on the 2008 studies, it would be worthwhile to now do a time course of ERa interactions with cytoplasmic/membrane partners (PI3K, Src, FAK, possibly other potential partners) using the PLA assay which should be more sensitive and provide an in situ assessment.

6. The authors use ERa interaction with the coregulator SRC3 and with p300 in the nucleus as a positive control for the PLA assay. These are known to be robust interactions. Can the authors compare in any way robustness of methyl-ERa interaction with its partners vs. that of ERa interaction with the coregulator SRC3 and with p300 in the nucleus ?

7. The Materials and Methods (p.11) are very minimally described, leaving many questions.
(a) State where the little known CLB-SAV cells were obtained. Why were these used instead of better known ER-positive cells such as ZR75 or T47D cells? (b) What are the relative ERa and mERa levels and ratios in MCF7 and CLB-SAV cells? (All that is stated is that these are both ER-positive breast cancer cells.)

8. Describe in Materials and Methods how the authors are performing the PLA assay even though the PLA assay is presented in ref.16 (which is not even referenced in Materials and Methods). Mention is only made to following the manufacturer's instructions. All antibodies used should also be listed and the PLA assay described, as used, since this is so critical to this study.

Minor Comments

10. Important relevant references dealing with ERa and c-Src interactions, such as by Joyce Slingerland, and on p130Cas interaction with ERa by Shupnik and Silva should be added.

Resubmission 31 May 2012

Point-by-point response to the reviewer's comments

We appreciate the overall comments from the reviewers and, we thank for their suggestions to improve the manuscript

Referee #1 >
> Better controls need to be included to confirm that PLA doesn't occur when two proteins are expressed in the same cell (i.e in the presence of tamoxifen, or between ER and a non-related growth factor).
The use of PLA for protein-protein interaction is novel and the application to study non-genomic interactions is a very nice way of using this technology. However, I'm still unsure how specific it is. In all cases, a series of dots are revealed which may still be non-specific interactions. The only time the dots are not seen is when one of the protein halves is missing (i.e. in ER negative cells, or after silencing ER). However, is it not possible that any proteins could be shown to be in proximity in a small fraction of the cells in a cell population? The addition of E2 does increase dot formation, but this could simply be due to the sequestration of ER by heat shock protein in untreated conditions. A much better control would be to repeat this experiment in the presence of an antagonist (such as tamoxifen), where ER is still present but is unlikely to interact with growth factors intermediates. In addition, it is essential that a non-interacting growth factor be tested (one that is expressed in the cells). Without this, we may be shown the few cell examples where protein aggregates have formed between numerous proteins, including ER and Src/PI3K.

As suggested by referre1, we have performed more controls to valid PLA technology in cells. We treated MCF-7 cells with E2 in the presence or in absence of tamoxifen and as expected we found a significant decrease in the numbers of dots for ER/PI3K and ER/Src. (See Figure S1)

We performed another control analysing FAK and ERα interaction. We have already demonstrated by COIP that FAK is present in the complex with Src /ERα and PI3K, and its recruitment is mediated via Src. (Le Romancer et al, Mol Cell 2008). Here we can see that although we detect red dots for the couple FAK/Src, we do not see a significant signal between FAK and ERα suggesting that the distance between the 2 proteins is too far to be able to detect any interaction (See figure S3)

- I am a little dubious about the data when only shown a couple of cells as examples. Can this data be quantitated across an entire cellular population and plotted graphically? Some of this is provided in Supplementary figures but should be included in the main figures.

It is true that on the figures we present only a few cells, but the counting plotted for each experiment is the mean of the number of dots for a 100 cells. In the new version of the manuscript, we quantified the signals for each experiment.

- Does the methylated ER recognise total ER? It seems unlikely that methylated ER exists in 66% of ER negative tumors. Is it not more likely that the antibody recognises a methyl group on another protein? Is there a control experiment that can be done to prove that this is specific......i.e. would siRNA to ER reduce the amount of detected methyl-ER?

Our antibody has already been extensively validated in our previous paper (Le Romancer et al, Mol Cell 2008). We have shown that the antibody does not recognize a methyl group (see supplemental data in the corresponding article).
In our new cohort of patients, we detect mERα with PLA using 2 different antibodies targeting ERα (HC-20 antibody and anti-mERα). The use of a pair of antibodies targeting the same protein gives a strong specificity to the obtained signal.
In this study, we found that methylated ERα is highly expressed in 56% of breast tumors and this is in accordance with what we published before (55% of hypermethylated ER in 164 breast tumor samples. In ERα negative tumors, we found 41% of the samples expressing a high level of mERα. Moreover, we detect also the

ERα/Src complex at the same frequency and this is performed by different antibodies validating the specificity of our results.

> **- I appreciate the desire to establish a new approach for defining ER negative tumors that may respond to treatments. However histological approaches use a very low cut-off (only 1% of cells need to be positive for ER to be called an ER positive tumor). If PLA is more sensitive, which I am happy to believe, then it needs to be shown that the PLA positive (histology negative) tumors or cell line models, respond to antiestrogens. If this doesn't happen then the whole concept is moot. It is well established that cohorts of ER negative tumors gain zero benefit from antiestrogen treatment, so if PLA can identify low level ER, yet the tumors gain no benefit from ER antagonists, I'm unsure of the benefit of using PLA. If the authors can find even a single cell line model that is ER negative by histology, but positive by PLA and responds to antiestrogens, I will be convinced.**
> - Similarly, the comment about triple negative tumour is redundant. There is no clinical benefit in triple negative tumors to ER antagonists. As such, it is unlikely that PLA will reveal a subpopulation that can gain any clinical benefit. If the authors can convince me that the clinical data and current paradigms are wrong, I would be willing to listen.
>
Maybe there is a misunderstanding for this point. We do not say that PLA is more sensitive to detect low level of ERα. This technology allows detecting an interaction between 2 proteins and in our case when ERα binds to Src and PI3K, it triggers a downstream activation as demonstrated by the correlation with activation of Akt (p-Akt). >

For the criticism about triple negative tumors, now in the new version of the manuscript, we extended our study on ERα- positive and negative- breast tumor samples. However, our results still suggest that detecting ER/src complex in ER-negative breast tumors could orientate the treatment towards anti-aromatase combined with Src inhibitosr.

> Referee #2 (Comments on Novelty/Model System):
>
> For the first time the authors visualise the estradiol-dependent ERalpha/PI3K/Src complex, which has a key role in regulating a proliferative signaling network. This complex is now found not only in hormone-responsive cell lines but also in normal human breast samples,human mammary cancer samples, adipocytes. The role of receptor methylation in regulating the complex assembly is demonstrated.The authors observe a correlation between the level of methylated receptor, but not the nuclear receptor, and the intensity of the complex in breast cancer.
> Interestingly, the majority of the analyzed breast tumors classified as ERalpha negative express the cytoplasmic complex implicating that the complex or the methylated receptor could be additional markers of breast cancer progression. The utility of these assays is also supported by the observed increase of axillary lymph node involvement with increased level of arginine methylated receptor.
>
>
>
> Referee #2 (Other Remarks):
>
> Use of a new method, the Proximity Ligation Assay, allows to clearly visualize in a variety of conditions and cell types a signaling complex which is specific for the nongenomic proliferative action of estradiol and requires ERalpha arginine methylation.Of remarkable clinical relevance is the presence of the complex in receptor negative human breast cancers and the increased expression of the modified receptor in tumors with increased axillary involvement. The report could be improved if some of the figures in the supplemental material are moved to the main text. This goal might be easily realized if this report will be published as a full research article. Alternatively, at least Fig S1 and S5 should be included in Fig 1B and D, respectively. The spelling of some authors in the text, but not in the references, is wrong.
>
We have included supplemental figure in the text and our manuscript is now presented as a regular paper and we have corrected the spelling of authors.

> Referee #3:
>
> Major Comments and Questions

> 1.Throughout the manuscript, the authors need to more quantitatively analyze the data and determine statistical significance. For example, based on data in Table 1, the authors talk about "high expression of methyl-ERa and expression levels of the complexes" (p.9 top), but this needs to be done with rigorous statistical assessments. The authors use a cut-off of less than 1 dot per cell (low) and more than 1 dot per cell (high), but is this most appropriate since dots per cell range from 0 to 10? Is there a gradation for more than 1 dot that could be evaluated? I think a better statistical analysis should be performed to determine is a correlation truly exists and what is its level of significance based on a rigorous analysis of the data.

➢ **2.The authors conclude on p. 9 top that "it appeared that the expression of methyl-ERa increased with the axillary lymph node involvement (Table S1)". But Table S1 is based on only 12 patients with lymph node metastasis. This is a small number of patients to be meaningful in drawing any conclusions. Also, more clinical information on the patients and tumors is needed. For example, number of nodes involved, not just positive or negative for nodes. And would be valuable to provide information on stage of the breast tumors and treatment the patients might have been receiving.**

➢ **> 3.Why do the authors (p.8 bottom and Table 1) study mERa (methyl-ERa) only in ER-negative breast tumors since mERa is found in a large fraction of ERa-positive breast tumors based on their prior 2008 publication? The authors now show ERa/Src and ERa/PI3K interaction by PLA assay in only 1 ER-positive tumor (Fig.2A) and in 1 ER-negative breast tumor (Fig.2B).**

We agree with the referee that our study was done on a small number of samples. To circumvent all these criticisms, we extended our cohort to 175 of breast tumors including ERα–positive and ERα–negative tumors.
➢ In the new manuscript, we performed real statistical analysis with robust tools. We found that high expression of ERα/Src/PI3K complex in the cytoplasm of breast tumors is a new biomarker.
➢
➢

> 4. What are the relative amounts of nuclear ERa and cytoplasmic mERa in the breast cancer cell lines studied (MCF7, CLB-SAV cells) and in the human mammoplasty samples, and in the human tumor specimens? There is no quantitation of this. I realize that this is not straightforward since different antibodies are used, but I think the authors should try to give some information about this in this manuscript.
➢
➢ We have already tried to address this point in cells quantifying the amount of methylated ERα immunoprecipitated compared with total ERα and it corresponds to a fraction of around 1% of total ERα.
➢ Within tumours it is variable as we can detect a lot of this complex even in ERα negative tumors (devoid of ERα in the nucleus).
➢

> 5. Interactions by PLA are done only at 1 time point, 5 minutes, as in their 2008 paper. Even though methylation and downstream events are said to be very transient, based on the 2008 studies, it would be worthwhile to now do a time course of ERa interactions with cytoplasmic/membrane partners (PI3K, Src, FAK, possibly other potential partners) using the PLA assay which should be more sensitive and provide an in situ assessment.
➢
➢ We performed a time course over 15 min of estrogen treatment (Figure 1) and analyzed ERα/SRc and ERα/PI3K interactions. For FAK, as we explained before, we were unable to detect interaction, with FAK by PLA experiments. The goal of this part was to validate the tools to study ERα/Src/PI3K interaction that is the main core of the complex, to move to study on primary samples.
➢

> **6. The authors use ERa interaction with the coregulator SRC3 and with p300 in the nucleus as a positive control for the PLA assay. These are known to be robust interactions. Can the authors**

compare in any way robustness of methyl-ERa interaction with its partners vs. that of ERa interaction with the coregulator SRC3 and with p300 in the nucleus ?

- For ERα/SRC3 or ERα/p300 interactions it is impossible to quantify the signals, as the dots are clamped. This was expected as these 3 proteins are mainly expressed in the nucleus. For cytoplasmic ERα we know that it concerns a small amount of ERα.

> 7. The Materials and Methods (p.11) are very minimally described, leaving many questions.
> (a) State where the little known CLB-SAV cells were obtained. Why were these used instead of better known ER-positive cells such as ZR75 or T47D cells? (b) What are the relative ERa and mERa levels and ratios in MCF7 and CLB-SAV cells? (All that is stated is that these are both ER-positive breast cancer cells.)

- As the referee requested, we gave more informations in the Material and methods sections. We also studied ERα/Src and ERα/PI3K interaction in other cell lines (Figure S4).

> 8. Describe in Materials and Methods how the authors are performing the PLA assay even though the PLA assay is presented in ref.16 (which is not even referenced in Materials and Methods). Mention is only made to following the manufacturer's instructions. All antibodies used should also be listed and the PLA assay described, as used, since this is so critical to this study.

- We gave more details about PLA assay and antibodies used for these kind of experiments.

- **> Minor Comments**

> 10. Important relevant references dealing with ERa and c-Src interactions, such as by Joyce Slingerland, and on p130Cas interaction with ERa by Shupnik and Silva should be added.

- As the referee suggested, we have included new references in the text.

2nd Editorial Decision 22 June 2012

Thank you for the submission of your manuscript to EMBO Molecular Medicine and please accept my apologies for the delayed response. We have now heard back from the three referees whom we asked to evaluate your manuscript. In addition, we consulted with a Senior Board Member and discussed the decision with our Chief Editor. Although the referees and the board member find the study to be of potential interest, they all raise a most important concern about the clinical significance of your study that we would like you to address in a final round of major revision.

As you will see from the reports below, while Referee #1 is now satisfied with the revision as most of his/her concerns have been addressed, Referees #2 and #3 are new referees and they raise again the clinical significance of the findings together with limited mechanistic insight.

I have to say that the mechanistic insight in this case would not be our principal concern should you be able to convincingly address the clinical relevance issue. Referee #2 does not believe that the issues raised by Referee #1 in the 1st round have been addressed. Referee #1 actually agreed with many of the other reviewers comments in the cross-commenting section we always perform at EMBO, mentioning that indeed the comment about Tamoxifen not working in (nuclear) ER negative patients was very relevant. This point was also strongly supported by our Board Advisor, who stated "The field would like to know if blocking the cytoplasmic ER complex impacts on e.g. proliferation. [...] there's no way a company would do another clinical trial with an endocrine agent in patients that don't have nuclear ER. Src inhibitors are in the clinic but it would be hard to base a Src trial on the ER-Src interaction described [here]".

Nevertheless, as the referees and our Advisor all agree on the potential interest of the study, we would like to give you another opportunity to address the clinical relevance issue as best as you possibly can, with the understanding that the other issues should also be addressed.

Please note that it is EMBO Molecular Medicine policy to allow only a single round of revision and that, as acceptance or rejection of the manuscript will depend on another round of review, your responses should be as complete as possible.

Revised manuscripts should be submitted within three months of a request for revision; they will otherwise be treated as new submissions, except under exceptional circumstances in which a short extension is obtained from the editor. Also, the length of the revised manuscript may not exceed 60,000 characters (including spaces) and, including figures, the paper must ultimately fit onto optimally ten pages of the journal. You may consider including any peripheral data (but not methods in their entirety) in the form of Supplementary information.

I look forward to seeing a revised form of your manuscript as soon as possible.

Yours sincerely,

Editor
EMBO Molecular Medicine

***** Reviewer's comments *****

Referee #1:

The authors have addressed my concerns.

Referee #2 (Comments on Novelty/Model System):

This is an interesting and potentially important study that employs an emerging new technology to address a controversial issue i.e. the role of rapid, ER -mediated cytoplasmic signaling in breast

cancer biology and treatment. The data are novel and of potential clinical utility. The study is based on biological experiments conducted in well characterized human breast cancer cell lines with extension of these findings to patient clinical material i.e. primary breast cancer pathology specimens. This is a well documented and widely utilized experimental paradigm for biomarker studies in breast cancer.

Referee #2 (Other Remarks):

This manuscript addresses a controversial and unresolved issue of the role of cytoplasmic, rapid estrogen signaling in breast cancer and its relationship to therapeutic responsiveness. The reviewer has no experience with PLA technology but if it is measuring what is claimed then the new data presented herein provide convincing evidence for ER /Src and ER /PI3K interactions in breast cancer cell lines and in breast cancer pathology specimens, albeit at a low frequency compared to the total pool of cellular ER . Many of the issues related to specificity and quantitation of the approach have been well addressed in response to the initial review including significant additional data. Thus, it is evident that these interactions exist and are modulated as would be expected from the known biology i.e. rapid time course, dependent on ER, down-regulated by Tam, Src and PI3K inhibitors. However, their relevance to therapeutic responsiveness and patient outcome is less clear.

The primary issue with the data are the studies on the 175 clinical specimens and their interpretation. Published data from the Oxford Overview meta-analysis of adjuvant endocrine therapy failed to provide evidence for meaningful responses to tamoxifen in ER negative patients. This begs the question what is the clinical relevance and potential clinical utility of the reported cytoplasmic interactions in ER negative breast cancers? An issue pertinent to this, and raised at the first review but not rebutted, is to test the effects of tamoxifen in an ER negative (by IHC) cell line that is PLA positive for ER cytoplasmic interaction with PI3K or Src. Irrespective of the outcome this would be an informative experiment and aid future testing of other endocrine therapies +/- PI3K and Src inhibitors in such phenotypes.

The clinical data are also difficult to interpret in the absence of information on treatment. Presumably ER positive patients received standard adjuvant endocrine therapy (AIs or tamoxifen?). If this is the case ER /Src and ER /PI3K might be markers of the luminal B endocrine resistant phenotype since Osborne and others have implicated cytoplasmic ER signaling in this phenotype. However, these correlations which were significant for DFS were not for OS. Thus, the authors conclusion that "our work strongly suggests that nongenomic signaling pathways should also be taken into account at diagnosis to orientate therapy" is probably premature and should be modified. Clearly these studies require independent validation in the context of randomized clinical trials of endocrine therapy where these parameters can be assessed retrospectively. While this is beyond the scope of the current study it is worthy of presentation and emphasis in the Discussion.

Finally, the claim of "ER /Src/PI3K protein complex potentially constitutes a new independent diagnostic biomarker" needs to be discussed in the context of this particular patient population where several classical prognostic markers e.g. ER/PR, grade and nodes are not significant suggesting some selection bias and again the need for independent validation.

In summary, this is an interesting and potentially important study that provides strong evidence for the formation of cytoplasmic ER signaling complexes in breast cancer. The potential relevance of these new data to therapeutic responsiveness and as clinically useful biomarkers of prognosis and endocrine resistance needs to be interpreted with more caution.

Referee #3 (Comments on Novelty/Model System):

Technical quality raing stems mainly from the difficult task of meaningfully and accurately quantifying the dots/interactions, determining high versus low levels, with no comments on single tumor heterogenity which there likely is. If the system was quantitatively reliable,and it could be adapted to clinical specimens this would be helpful to classifying breast cancer.

Referee #3 (Other Remarks):

I think the technique if more rigorously established to quantify
the interactions and presence of cytoplasmic ERalpha/Src would be a contribution as justification for publication, but the correlative output data don't seem strong and convincing to me, in this manuscript. Further, there are no additional insights as to methylation of ER and signaling by ER outside the nucleus, beyond what is known and published from several labs.

Poulard et al present an assessment of methylated ERalpha (mER) in the cytoplasm of breast cancer cell lines and patient samples, and show association of mER with Src and PI3K using a newer approach/technique, proximity ligation assay (PLA). The authors then correlate their findings to patient characteristics and outcomes to support the idea that rapid signaling by the non-nuclear receptors could contribute to more aggressive tumor biology in both ER positive and ER negative tumors (the latter presumably where nuclear ER alpha was not detected).

1. The data build upon the previously published work of the authors, showing that methylation of ERalpha by PRMT1 (arginine methyltransferase) exists in breast cancer specimens, and results in cytoplasmic association of ER with the signaling molecules, and that methylated ER is necessary for AKT phosphorylation. The authors do not extend their molecular studies to determine more about methylation and why it causes productive signaling, mechanistically. It already has been established from the papers of Aurrichio and from Miguel Beato, that ER-Src-PI3K are in a complex at the membrane/cytoplasm interface, and they each showed that progesterone receptor is part of that complex, but that is not shown by the authors of this paper (appears not to be rigorously investigated). Thus the paper is mainly to establish the presence of this complex in human breast cancer cell lines and specimens.
2. Since there are many potential important signals downstream of cytoplasmic/membrane ER (and several of their Figures (see Fig 1Aa and b) suggest clear membrane localization but this is not considered strongly), the authors could have established whether methylated ER is needed for a few or many signals, important to the biology of estrogen action. How does E2 induce methylation of ERalpha? Work from the Marino and Levin labs has established that membrane ER is the receptor that signals through many pathways in breast cancer, and palmitoylation drives the receptor to the membrane to contact and activate many G protein subunits for downstream signaling. This concept is not integrated with methylation here. Thus, there are no advances in the cellular biology of this receptor in the current manuscript.
3. It has recently been published by the Rimm lab that of ~3200 breast cancer specimens, immuno-fluorescent microscopy detects membrane ER in about 1.5% (Clin Canc Res , 2012). The low levels may be due to sensitivity of their assay limitations, but the principle is now established that membrane/cytoplasmic ERalpha exists in breast cancer specimens. The technique used by Poulard et al here could be an advance in this respect, but adaptation to the constraints of a clinical lab would need to be accomplished. The use of the exogenous pY-ERalpha peptide that competes off Src/Er or Src/PI3K association (Figure 2) is not clear as to how it gets into the cell to promote this competition.
4. The clinical correlate data has several limitations. Several of the associations are quite modest, though statistically significant because of the high number of tumor samples in the studies (see Table 2). Several Tables (Table S1 for instance) are not well explained in the Fig legend so it is hard to know what this means. There are many comparisons made but the correlations of ER/Src with clinical parameters seems difficult to understand from a common theme of aggressiveness because other parameters in this regard show no association. Also, it is difficult to quantitate the dots, and this quantitation as related to clinical outcomes, especially as a function of "high" versus "low" ERa/Src or ERa/PI3K (how is that rigorously determined as imaging software is often subjective based upon the field chosen). Is there tumor heterogeneity that make interpretation difficult within the same tumor-this is not mentioned. Finally, delineating ER negative tumors (presumably nuclear ER negative) is not well developed and thus the claims of expression of cytoplasmic ET/Src seems a little premature, stating more than half these tumors show this pattern, and what this means for patient outcomes.

1st Revision - Authors' Response 14 August 2012

Replies to the referee's comments:
First of all, we would like to thank the Referees for their kind appreciation of our work, and for helpful suggestions that helped indeed to improve its clarity and impact. Point-by-point replies to the criticism raised are detailed below.

Referee #2

The primary issue with the data are the studies on the 175 clinical specimens and their interpretation. Published data from the Oxford Overview meta-analysis of adjuvant endocrine therapy failed to provide evidence for meaningful responses to tamoxifen in ERα negative patients. This begs the question what is the clinical relevance and potential clinical utility of the reported cytoplasmic interactions in ERα negative breast cancers? An issue pertinent to this, and raised at the first review but not rebutted, is to test the effects of tamoxifen in an ERα negative (by IHC) cell line that is PLA positive for ER cytoplasmic interaction with PI3K or Src. Irrespective of the outcome this would be an informative experiment and aid future testing of other endocrine therapies +/- PI3K and Src inhibitors in such phenotypes.

Response- It is true that the Oxford Overview does not advocate at all for the use of endocrine therapy for ER negative tumours in the adjuvant setting. However, we hypothesize that treatment combining endocrine therapy plus an agent that targets PI3K or Src may be of particular interest in the metastatic setting, especially if cancer cells express the ER/PI3K or ER/Src complexes. It would indeed have been interesting to test that in vitro, however we did not find so far such cell lines (expressing ERa exclusively in the cytoplasm). So, to answer to this important question, we planned to use mice engrafted with human breast tumours from patients (Marangoni and coll 2007). We have already screened these mice tumours for ERa /Src expression and we found several mice with tumours that do not express ERa in the nucleus, but express ERa/Src in the cytoplasm of tumour cells. We will use these models to test the effects of tamoxifen combined with kinase inhibitors, such as PI3K/mTor inhibitors or Src inhibitors. This is a full on-going project that will take place before setting up a clinical trial.

The clinical data are also difficult to interpret in the absence of information on treatment. Presumably ER positive patients received standard adjuvant endocrine therapy (AIs or tamoxifen?).

Response : Indeed the large majority of ER positive patients did receive adjuvant endocrine therapy (90% received tamoxifene because this cohort of patients was treated before 2000). The samples analyzed are primary tumors taken before treatment.

If this is the case ERα/Src and ERα/PI3K might be markers of the luminal B endocrine resistant phenotype since Osborne and others have implicated cytoplasmic ER signalling in this phenotype. However, these correlations which were significant for DFS were not for OS. Thus, the authors conclusion that "our work strongly suggests that nongenomic signalling pathways should also be taken into account at diagnosis to orientate therapy" is probably premature and should be modified.

Response : We agree. The sentence reworded is: "Our work suggests that the nongenomic signaling pathway may be taken into account to optimize targeted therapies"

Clearly these studies require independent validation in the context of randomized clinical trials of endocrine therapy where these parameters can be assessed retrospectively.

Response : We have included this statement in the discussion on page 14.

While this is beyond the scope of the current study it is worthy of presentation and emphasis in the Discussion.

Response : Again, we agree and we modified the last two paragraphs and the conclusion.

Finally, the claim of "ERα/Src/PI3K protein complex potentially constitutes a new independent diagnostic biomarker" needs to be discussed in the context of this particular patient population where several classical prognostic markers e.g. ER/PR, grade and nodes are not significant suggesting some selection bias and again the need for independent validation.

Response : We have included a sentence to clarify the fact that "our patient population was small and usual prognostic factors were not found to be significant in the current analysis". It definitively requires validation studies: we emphasize this in the revised version of the discussion..

Referee #3

1. The data build upon the previously published work of the authors, showing that methylation of ERalpha by PRMT1 (arginine methyltransferase) exists in breast cancer specimens, and results in cytoplasmic association of ER with the signalling molecules, and that methylated ER is necessary for AKT phosphorylation. The authors do not extend their molecular studies to determine more about methylation and why it causes productive signalling, mechanistically. It already has been established from the papers of Aurrichio and from Miguel Beato, that ER-Src-PI3K are in a complex at the membrane/cytoplasm interface, and they each showed that progesterone receptor is part of that complex, but that is not shown by the authors of this paper (appears not to be rigorously investigated). Thus the paper is mainly to establish the presence of this complex in human breast cancer cell lines and specimens.

Response-
It is true that knowing more about the regulation of ER methylation is an important challenge, and we are actually running projects treating this aspect. However, this is not the scope of the present work that focuses the study to the core of the complex ER/Src/PI3K in breast samples.

2. Since there are many potential important signals downstream of cytoplasmic/membrane ER (and several of their Figures (see Fig 1Aa and b) suggest clear membrane localization but this is not considered strongly), the authors could have established whether methylated ER is needed for a few or many signals, important to the biology of estrogen action. How does E2 induce methylation of ERalpha? Work from the Marino and Levin labs has established that membrane ER is the receptor that signals through many pathways in breast cancer, and palmitoylation drives the receptor to the membrane to contact and activate many G protein subunits for downstream signalling. This concept is not integrated with methylation here. Thus, there are no advances in the cellular biology of this receptor in the current manuscript.

Response- Maybe some interactions take place in the membrane in cell lines, but we have not performed a membrane staining to confirm this point. However, in primary tumours, it does not seem to us that ER/Src or ER/PI3K interactions are localized in the membrane. At this stage, we have not investigated a crosstalk between ERa palmitoylation with methylation.

3. It has recently been published by the Rimm lab that of ~3200 breast cancer specimens, immuno-fluorescent microscopy detects membrane ER in about 1.5% (Clin Canc Res , 2012). The low levels may be due to sensitivity of their assay limitations, but the principle is now established that membrane/cytoplasmic ERalpha exists in breast cancer specimens. The technique used by Poulard et al here could be an advance in this respect, but adaptation to the constraints of a clinical lab would need to be accomplished.

Response:
We are also concerned by this point, and actually, we have already introduced the PLA technique in our translational team which is part of the department of biopathology in Centre Léon Berard. We had to adapt the protocol to clinical constraints and perform the technique in 2 days instead of 1 day. Our technicians are able to perform it and we obtained results which are similar to those from the research team. We are also setting software that will perform the counting of the dots as well as the nuclei of tumoral cells.

4. The use of the exogenous pY-ERalpha peptide that competes off Src/Er or Src/PI3K association (Figure 2) is not clear as to how it gets into the cell to promote this competition.

Response:
The peptide pY-ERapla has already been used and validated in the paper from Varricchio and coll (Mol cancer research, 2007). They have shown that the peptide enters within MCF-7 cells independently of temperature, suggesting that it did not depend on energy.

5.The clinical correlate data has several limitations. Several of the associations are quite modest, though statistically significant because of the high number of tumour samples in the studies (see Table 2)..

Response: We agree and we have discussed in the revised version of the article that these clinical data have to be validated in independent cohorts ("Independent validation is required and this can be done in the context of randomized clinical trials with endocrine therapy where the estrogen nongenomic signaling can be assessed retrospectively" page 14; "This concept has to be validated in large prospective clinical studies" page 14).

6.There are many comparisons made but the correlations of ER/Src with clinical parameters seems difficult to understand from a common theme of aggressiveness because other parameters in this regard show no association.

Response: We agree and again we admit that the patient population has to be analyzed with caution since it is a retrospective analysis of a small population. A statement has been added to the discussion on page 14

6. Several Tables (Table S1 for instance) are not well explained in the Fig legend so it is hard to know what this means

We have clarified the figure legends of the Tables as requested.

7. Also, it is difficult to quantitate the dots, and this quantitation as related to clinical outcomes, especially as a function of "high" versus "low" ERa/Src or ERa/PI3K (how is that rigorously determined as imaging software is often subjective based upon the field chosen). Is there tumour heterogeneity that make interpretation difficult within the same tumour-this is not mentioned. Response:

Response:
We have done the analysis on TMAs blocks which include 3 cores of 600 microns for each tumour. In fact, each core is obtained in a different area of the tumour so that we try to take in account tumour heterogeneity. Furthermore, the high number of tumours that can be analysed using TMA usually overwhelms tumour heterogeneity. This approach (using triplicates cores) has been validated for TMA analysis of breast cancers using immunohistochemistry and FISH. For PLA assessment, we make an average of the staining while counting the 3 cores. We do not choose focal areas on the cores. In the vast majority of our cases, a homogenous staining within the 3 cores was observed except for 10 tumours. In these cases, the average of staining obtained from the 3 cores was used.

8.Finally, delineating ER negative tumours (presumably nuclear ER negative) is not well developed and thus the claims of expression of cytoplasmic ET/Src seems a little premature, stating more than half these tumours show this pattern, and what this means for patient outcomes.

Response:
In France, according to the national recommendations, tumours exhibiting less than 10% of ER positive cells qualify for ER negative tumours. In our data base, tumours with more than 1% and less than 9% of ER+ cells are rare, 2 to 3% of all the tumours. This point has been added in methods p17.

The fact that ER/Src expression is measured in more than half of the tumours is concordant with our previous study showing that more than half of breast tumours express methylated ER in the cytoplasm independently of the presence of ER in the nucleus. (Le Romancer et al, 2008).

Regarding the outcomes, we have noted in this paragraph of the discussion that the results were not statistically significant. However, we added a sentence pointing out the important fact that the expression of ERα/Src/PI3K complex may become a new target for ER negative tumors.

3rd Editorial Decision 28 August 2012

Thank you for the submission of your revised manuscript "Activation of rapid estrogen signaling in aggressive human breast cancers" to EMBO Molecular Medicine. We have now received the report from the reviewer who was asked to re-review your manuscript.

You will be glad to see that the reviewer is now globally supportive and we can proceed with official acceptance of your manuscript pending the minor changes detailed below:

- Please address the remaining concern raised by the reviewer.

- For experiments involving human subjects/material the submission must include a statement that informed consent was obtained from all subjects and that the experiments conformed to the principles set out in the WMA Declaration of Helsinki [http://www.wma.net/en/30publications/10policies/b3/] and the NIH Belmont Report [http://ohsr.od.nih.gov/guidelines/belmont.html]. Please see our Guide to Authors for further information and provide the necessary information in the respective Material and Methods part.

- The description of all reported data that includes statistical testing must state the name of the statistical test used to generate error bars and P values, the number (n) of independent experiments underlying each data point (not replicate measures of one sample), and the actual P value for each test (not merely 'significant' or 'P < 0.05').

- In addition, we noted that the labeling of some graphs (for example in Fig 1) is very small and will be hard to read in the final PDF. Please increase the font size accordingly.

I look forward to reading a new revised version of your manuscript as soon as possible.

Yours sincerely,

Editor
EMBO Molecular Medicine

***** Reviewer's comments *****

Referee #3 (Comments on Novelty/Model System):

It has been controversial as to whether membrane/cytoplasmic estrogen receptors are present and relevant to human breast cancer. The authors nicely show with a novel approach, the formation of a Src/ER/PI3K complex in cytoplasm, near the membrane, and correlate this to various parameters in human breast cancer specimens. The latter are somewhat interesting, although there are many limitations to this, as they admit. But the concept is very important, the data very convincing, and I believe justifies strong consideration for publication.

The paper could be published as a short report, but the clinical correlations probably require the longer format.

Referee #3 (Other Remarks):

The only thing I would ask further of the authors is to cite and briefly discuss and compare their findings to those recently published by Welsh et al. Clin Can Res18:118-126, 2012, where these authors found membrane-localized ER in 1.5% of breast cancer specimens (3200 patient sanples used). I think the paper by Poulard et al. here is a step further as a more sensitive assay for extra nuclear ER, but the authors should compare their findings and hence the limitations of the Welsh paper approach.

2nd Revision - Authors' Response 04 September 2012

I wish to thank you for your positive response. We have included as requested the patients statement about informed consent in the method section, specified the statistical informations and increased the font size of the graphs.

As requested by the Referee 3, we have cited the paper of Welsh and discussed their findings with ours. (Discussion section, page 12). ìThe analysis of ER /Src interaction in 175 breast tumor samples showed that 55% of breast tumors highly express this couple (Table 2). Our result is different from those of Welshís paper {Welsh, 2012 1239 /id}. They analyzed ER cytoplasmic expression by quantitative immunofluorescence on 3200 tumor samples and found that only 1.5% of tumors express cytoplasmic ER . This discrepancy is probably due to a lack of sensitivity, and comfort the use of the PLA technology as a powerful tool to measure estrogen non genomic signaling.î

I hope that you will judge our new version of the manuscript suitable for publication.

© EMBO
