# Supplementary material for: Activation of rapid oestrogen signalling in aggressive human breast cancers
Source: EMBO Mol Med. 2012 Oct 15;4(11):1200–13. doi: 10.1002/emmm.201201615 (PMC3494876; doi:10.1002/emmm.201201615)
Supplement: Supplementary file 2 [file emmm0004-1200-SD2.pdf]

Supplemental Data

Activation of rapid estrogen signaling in aggressive human breast cancers

Coralie Poulard, Isabelle Treilleux, Emilie Lavergne, Katia Bouchekioua-Bouzaghou, Sophie Goddard-Léon, Sylvie Chabaud, Olivier Trédan, Laura Corbo and Muriel Le Romancer

Table of contents

Figure S1: In Situ PLA detection of ERα/PI3K and ERα/Src interactions upon tamoxifen treatment

Figure S2: Detection of endogenous interactions between ERα/SRC3 and ERα/p300.

Figure S3: In Situ PLA detection of FAK/Src and FAK/ ERα interactions in MCF-7 cells

Figure S4: In situ PLA detection of endogenous ERα/PI3K and ERα/Src interactions in human breast cancer cell lines

Figure S5: Kaplan-Meier estimates of OS by ERα/Src expression groups.

Figure S6: Kaplan-Meier estimates of OS by ERα/PI3K expression groups

Figure S7: Kaplan-Meier estimates of patient's outcome for mERα/ERα expression groups.

Figure S8: Kaplan-Meier estimates of DFS by ERα/Src intensity groups

Table S1: Distribution of ERα/Src, ERα/PI3K and ERα/mERα data

Table S2: Distribution of clinical parameters according to groups of ERα/mERα expression

Table S3: **Sample description**: **Distribution of clinical parameters.**

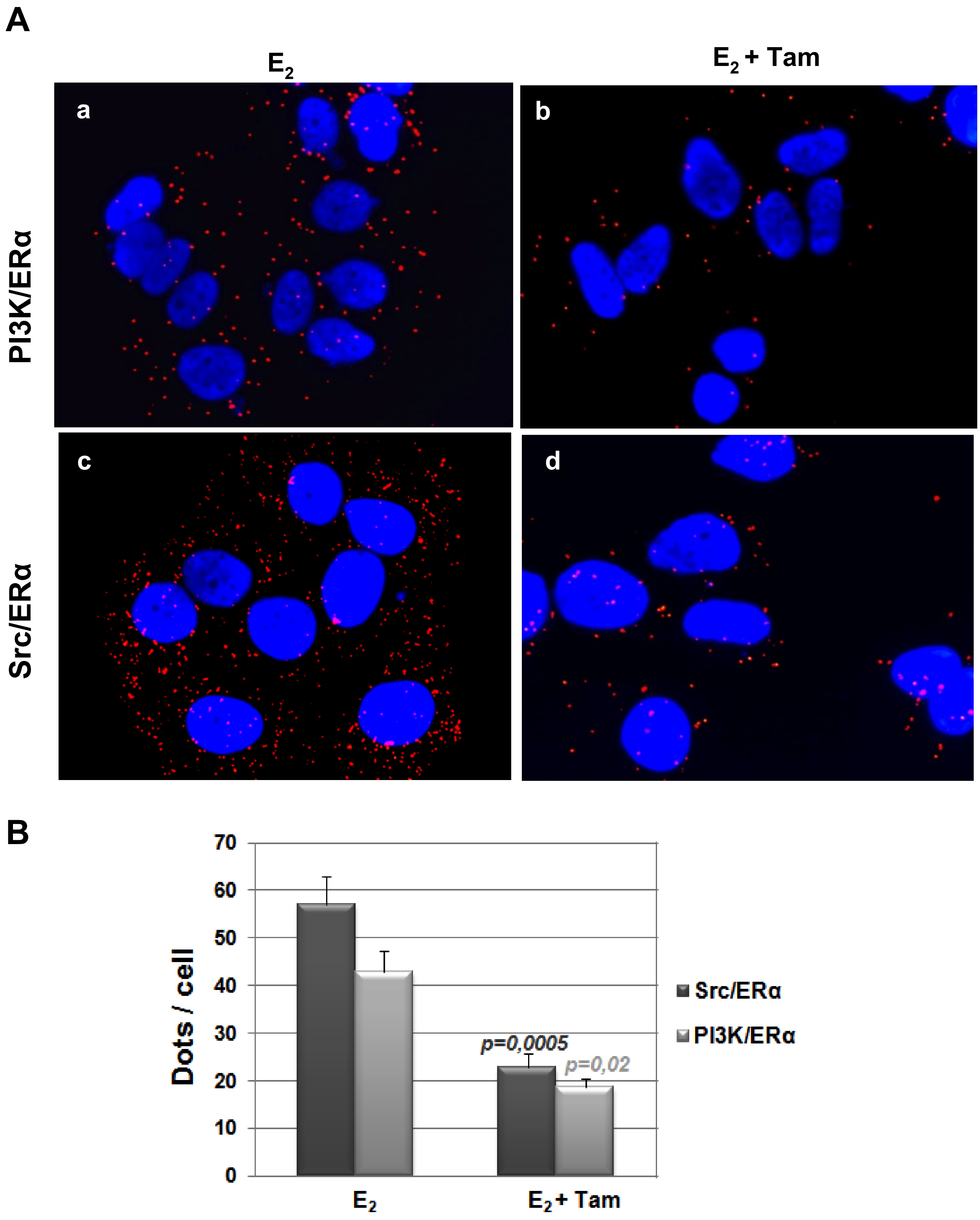

Figure S1: In Situ PLA detection of ERα/PI3K and ERα/Src interactions upon tamoxifen treatment. **A.** Estrogen-deprived MCF-7 cells were incubated with E_2 (10^{-8}M) or both E_2 plus tamoxifen ($5X10^{-6}$M) for 5 min. After fixation, *in situ* PLA for ERα/PI3K (panel a,b) and ERα/Src dimers (panel c,d) was performed with ERα-, Src-, and PI3K-specific antibodies. The nuclei were counterstained with DAPI (blue). **B.** Quantification of the number of signals per cell was performed by computer-assisted analysis as reported in the Materials and methods. The mean +/- s.e.m. of four experiments is shown. P-value was determined by Student's t-test.

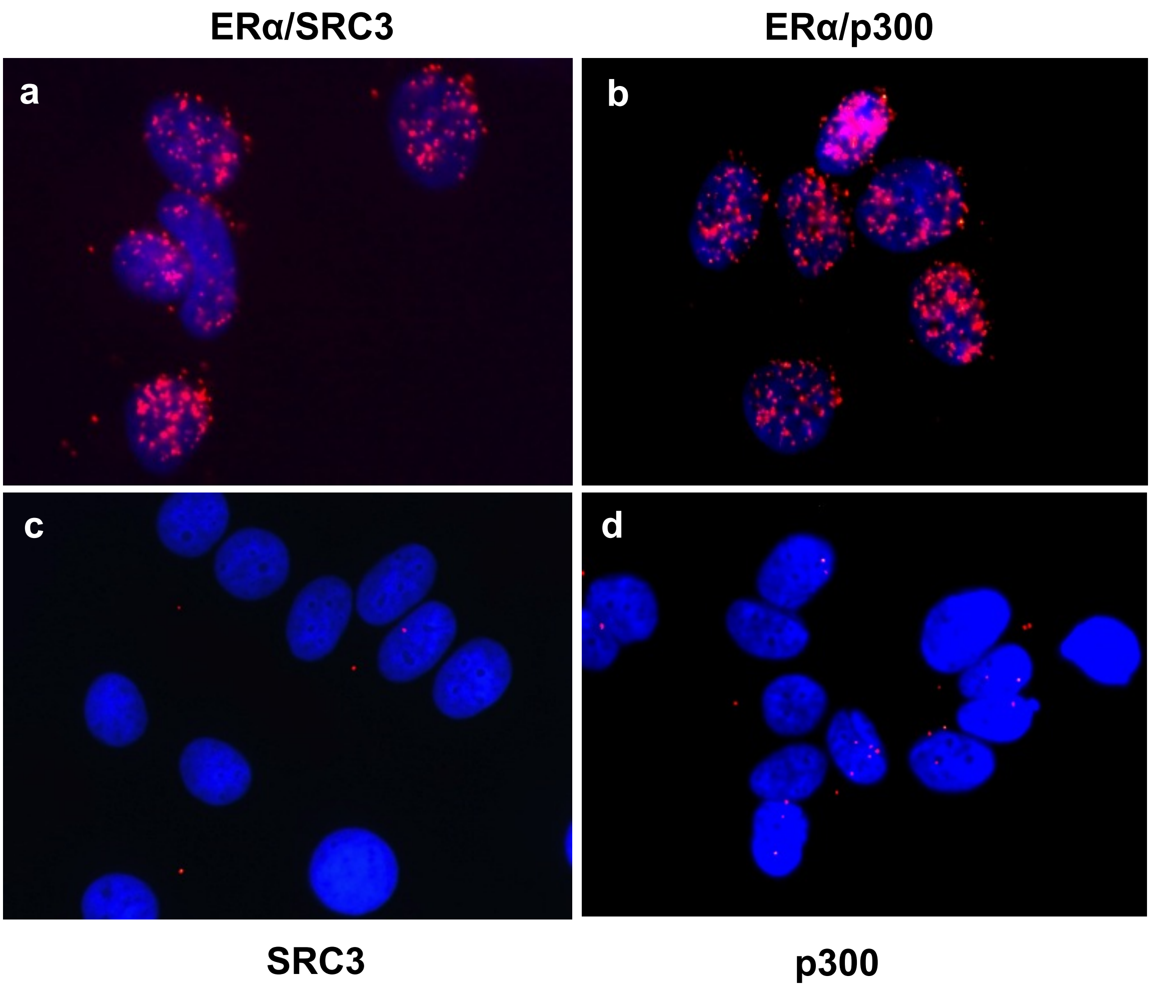

Figure S2: Detection of endogenous interactions between ERα/SRC3 and ERα/p300. PLA was performed on MCF-7 cells with two couples of antibodies: ERα/SRC3 (panel a) and ERα/p300 (panel b). Control experiments were performed using only one primary antibody: anti-SRC3 (panel c) or anti-p300 (panel d). The nuclei were counterstained with DAPI (blue).

A

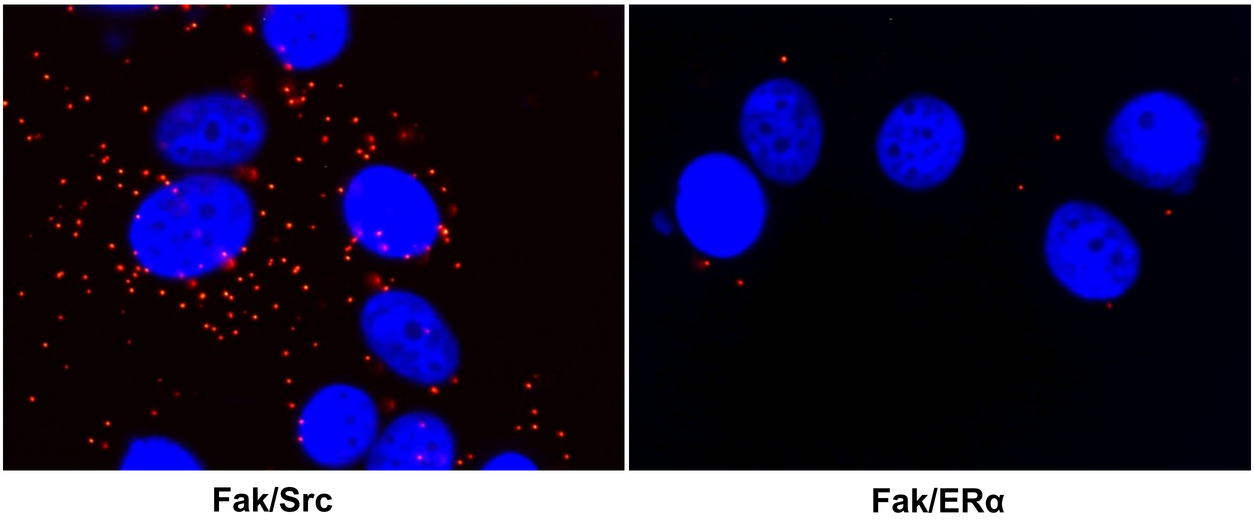

B

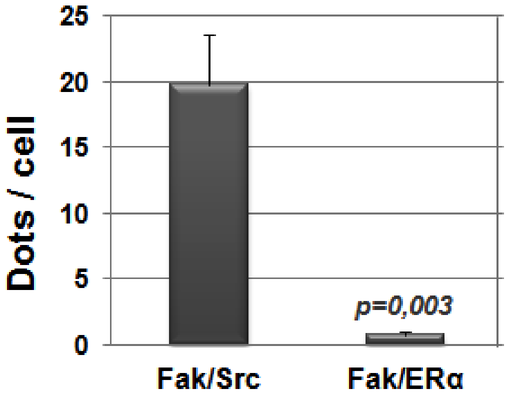

Figure S3: In Situ PLA detection of FAK/Src and FAK/ ERα interactions in MCF-7 cells
A. Estrogen-deprived MCF-7 cells were incubated with E_2 10^{-8}M for 5 min. After fixation, *in situ* PLA for FAK/Src (panel a) and FAK/ERα dimers (panel b) was performed with indicated antibodies. **B.** Quantification of the number of signals was performed as described previously. . The mean +/- s.e.m. of four experiments is shown. P-value was determined by Student's t-test.

A

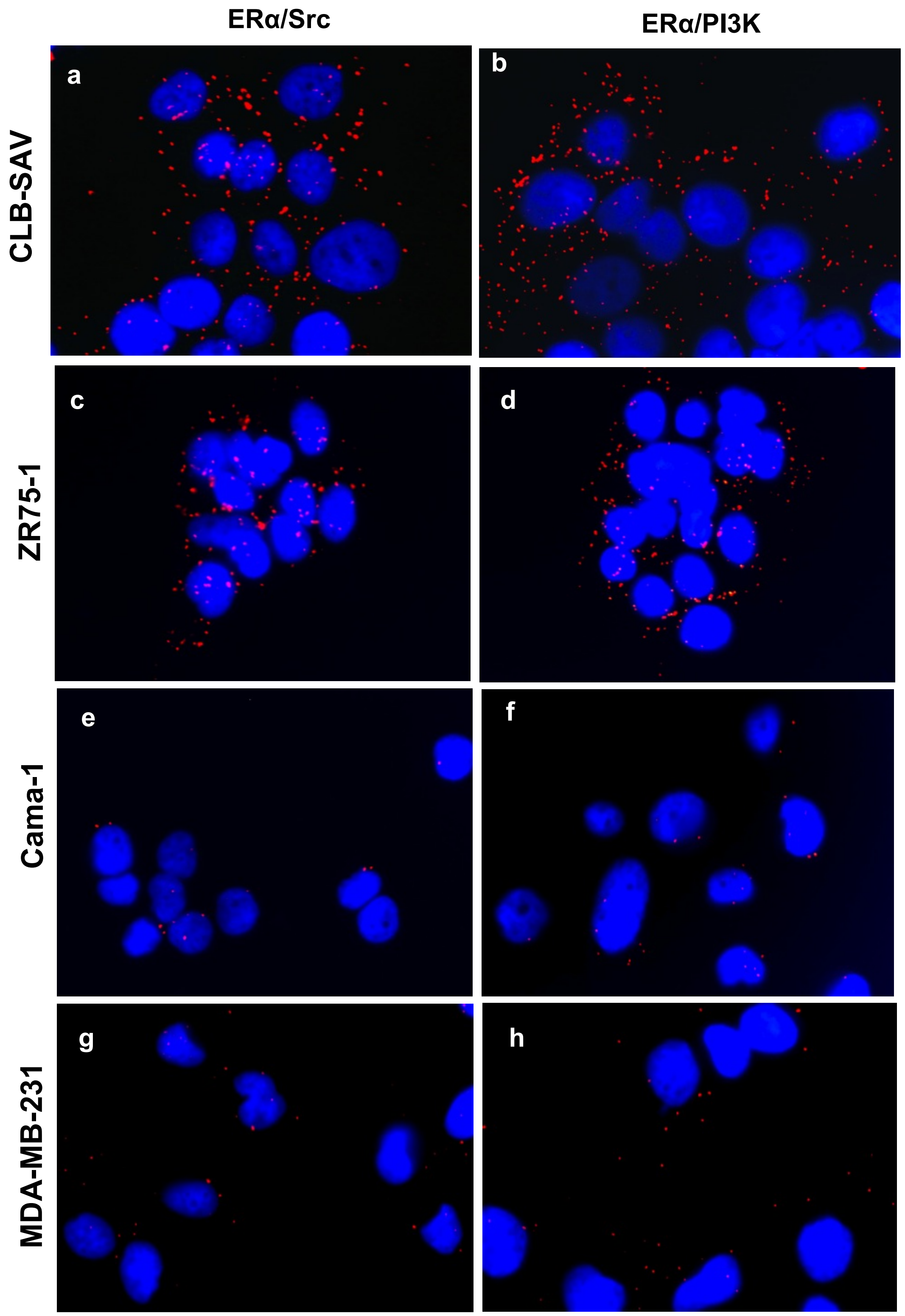

Figure S4

B

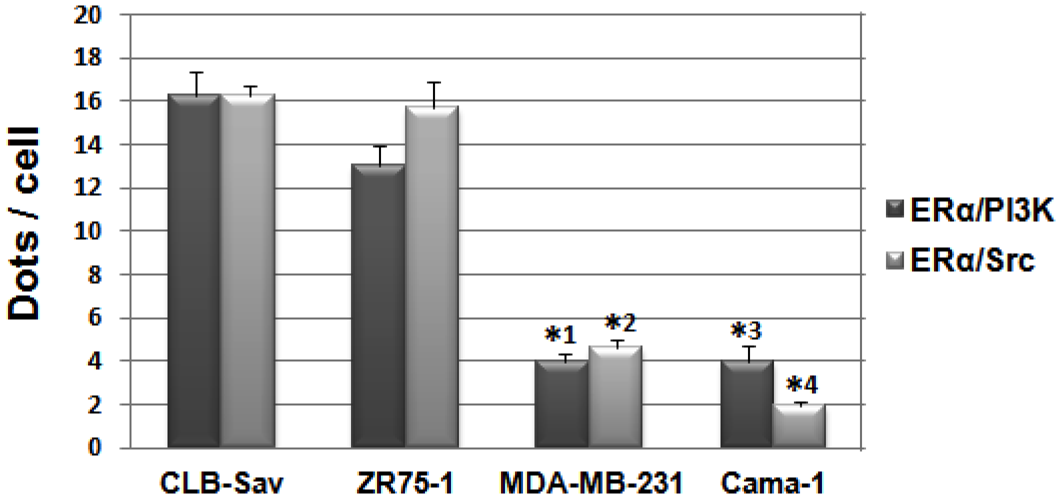

C

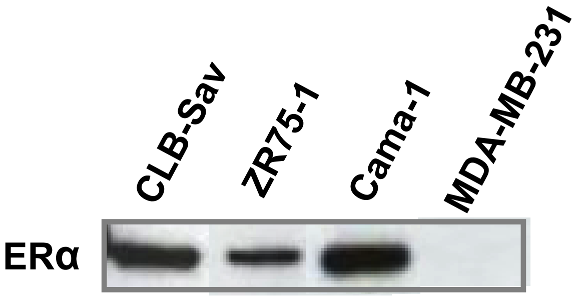

D

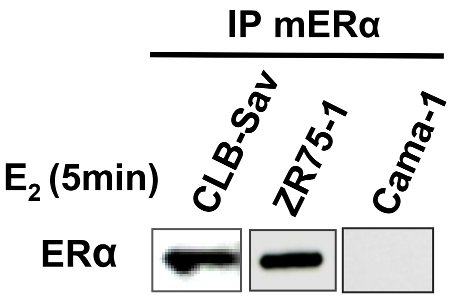

Figure S4: In situ PLA detection of endogenous ERα/PI3K and ERα/Src interactions in human breast cancer cell lines. A. *In situ* PLA of ERα/PI3K and ERα/Src interactions were performed in several human breast tumor cell lines. CLB-SAV (panels a,b); ZR75-1 (panels c,d); Cama-1 (panels e,f); MDA-MB-231 (panels g,h). **B.** Quantification of the number of signals was performed as described previously. The mean +/- s.e.m. of four experiments is shown. P-value was determined by Student's t-test. For PI3K/ERα : *1 p=0,0006 (CLB-Sav vs MDA-MB-231), p=0,0001 (ZR-75.1 vs MDA-MB-231), *3 p=1,6.10^{-5} (CLB-Sav vs Cama-1), p=0,0005 (ZR-75.1 vs Cama-1). For Src/ERα : *2 p=0,004 (CLB-Sav vs MDA-MB-231), p=0,0002 (ZR-75.1 vs MDA-MB-231), *4 p=0,004 (CLB-Sav vs Cama-1), p=0,0002 (ZR-75.1 vs Cama-1) **C.** Lysates of CLB-SAV, MDA-MB-231, ZR75-1 and Cama-1 cells were analyzed by western blot for ERα expression. **D.** Cells lysates were immunoprecipitated with anti-mERα and blotted with anti-ERα.

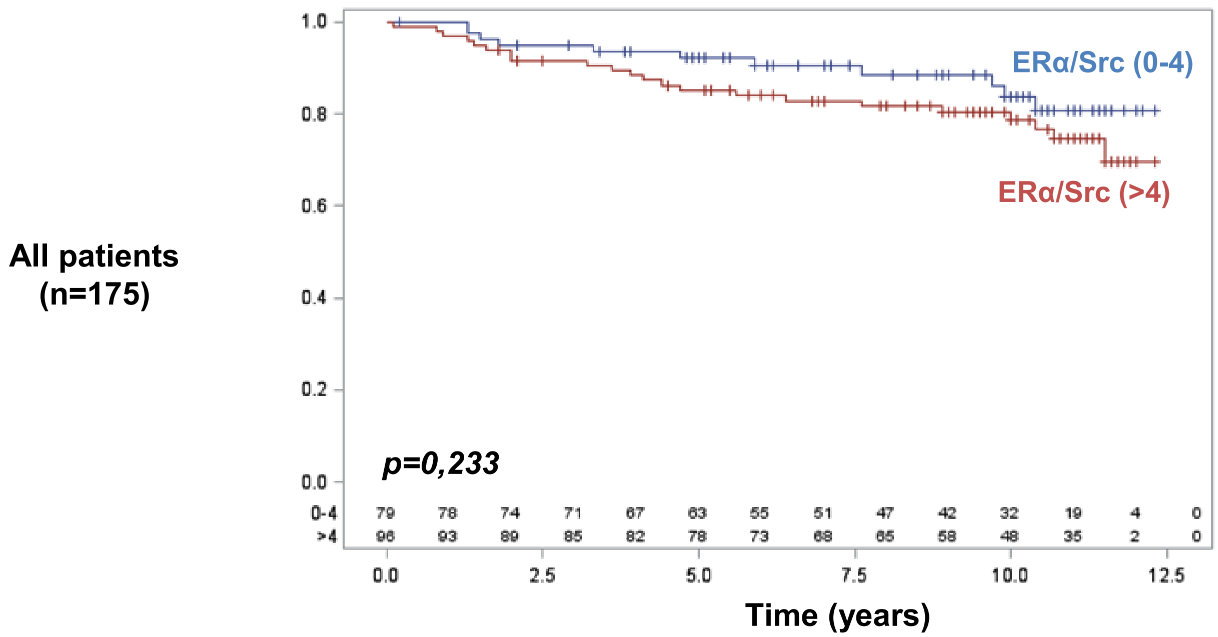

Figure S5: Kaplan-Meier estimates of OS by ERα/Src expression groups. Global population (with a cut off at 4 spots per cell).

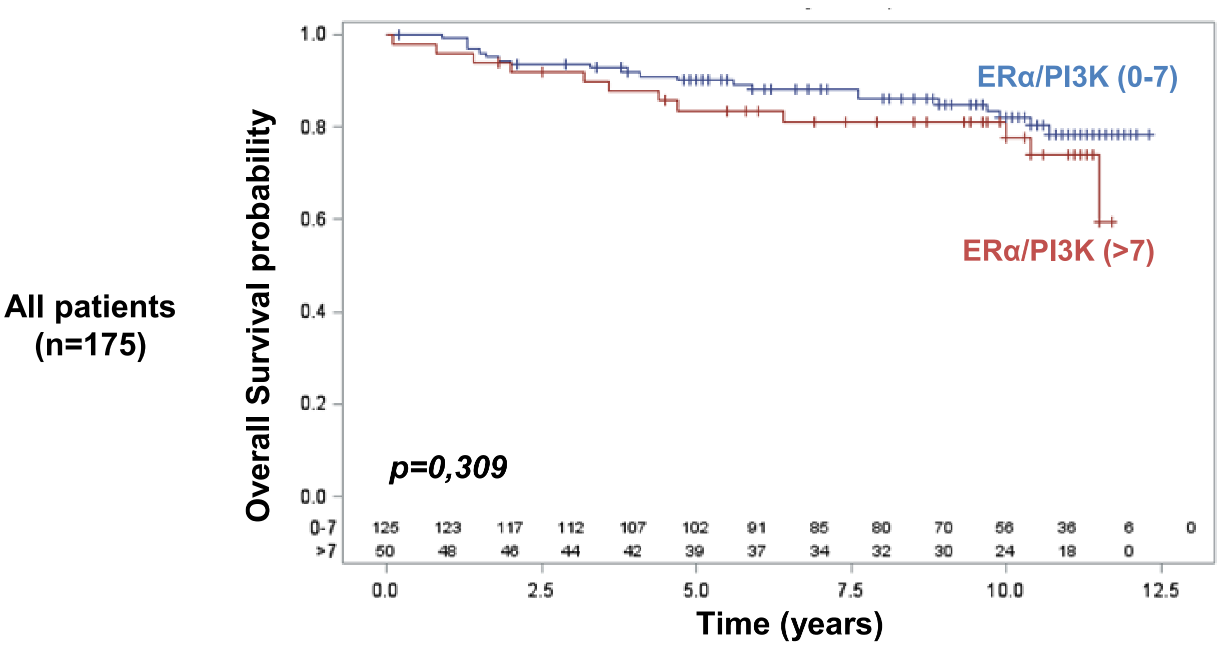

Figure S6: Kaplan-Meier estimates of OS by ERα/PI3K expression groups. Global population (with a cut off at 7 spots per cell).

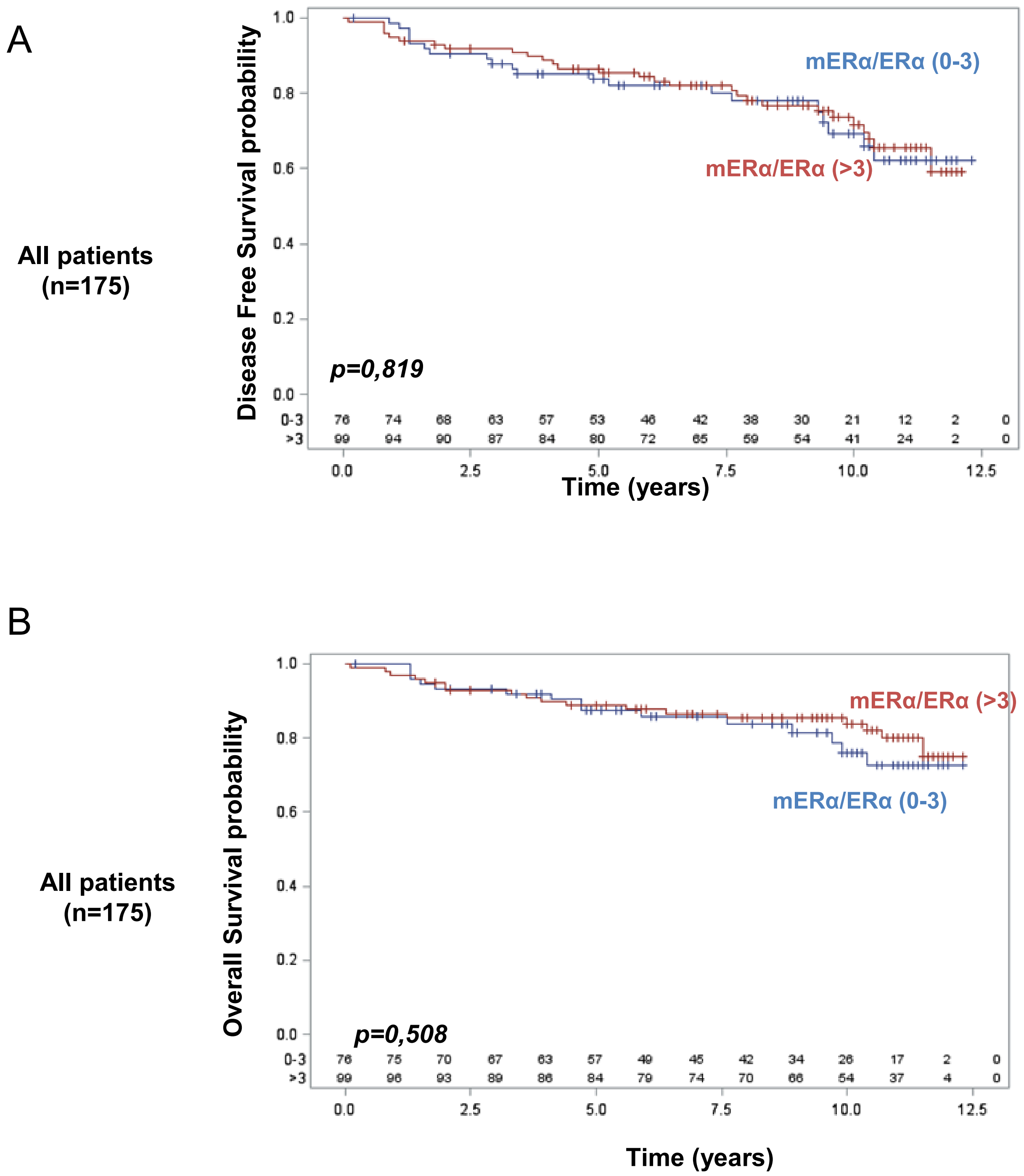

Figure S7: Kaplan-Meier estimates of patient's outcome for mERα/ERα expression groups. Global population (with a cut off at 3 spots per cell) for DFS (A) and for OS (B).

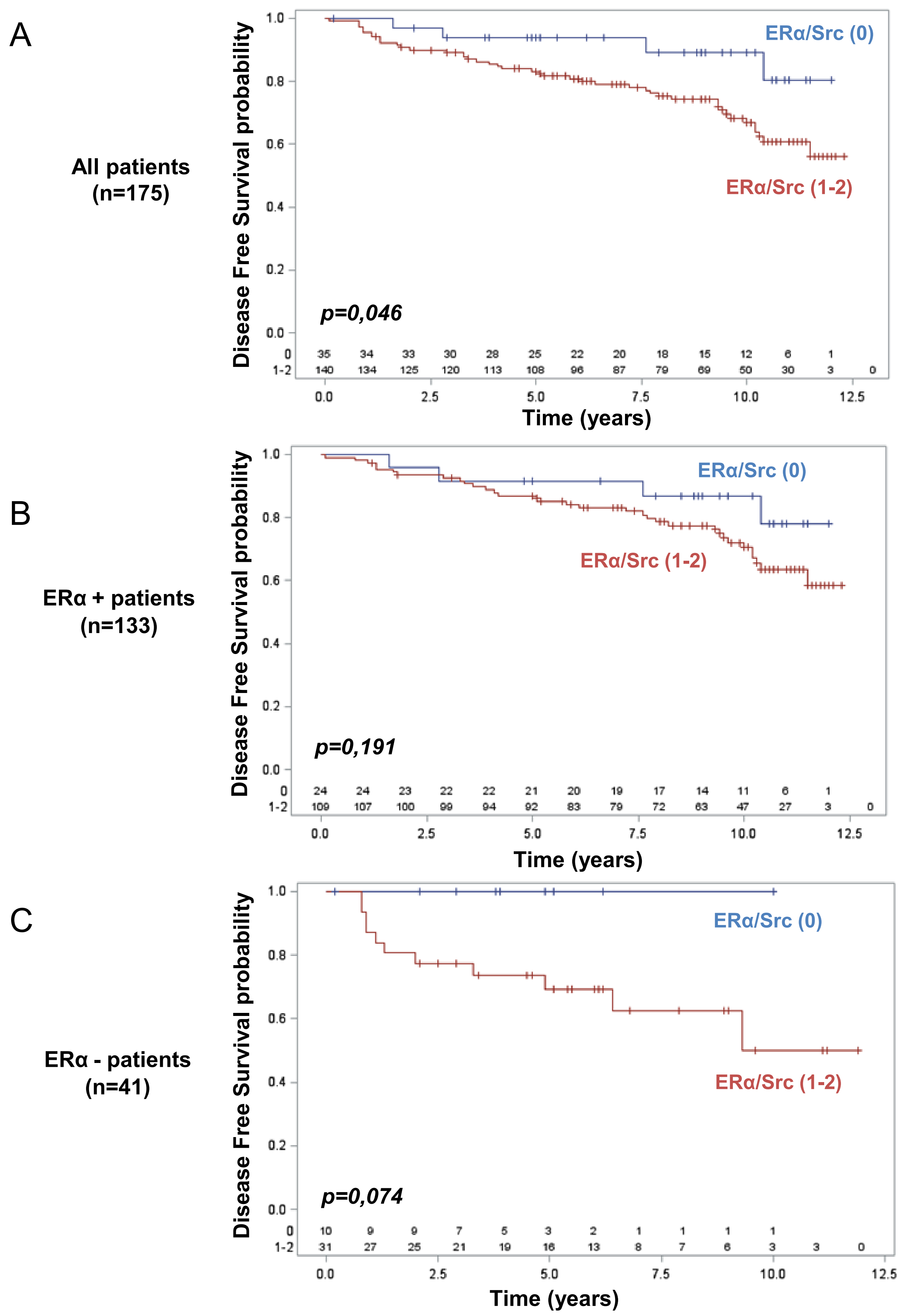

Figure S8: Kaplan-Meier estimates of DFS by ERα/Src intensity groups. A) Global population (with a cut off at 0 versus 1, 2). B) ERα -positive cases. C) ERα -negative cases.

Duolink counting (dots/cell)	Src/ERα		PI3K/ERα		mERα/ERα	
	N	%	N	%	N	%
0	4	2.3	10	5.7	14	8.0
1	27	15.4	17	9.7	26	14.9
2	9	5.1	4	2.3	16	9.1
3	16	9.1	16	9.1	20	11.4
4	23	13.1	18	10.3	25	14.3
5	22	12.6	26	14.9	26	14.9
6	17	9.7	18	10.3	11	6.3
7	15	8.6	16	9.1	15	8.6
8	18	10.3	12	6.9	10	5.7
9	9	5.1	12	6.9	3	1.7
10	5	2.9	6	3.4	3	1.7
11	5	2.9	8	4.6	2	1.1
12	1	0.6	2	1.1	1	0.6
13	2	1.1	3	1.7	0	
14	0		2	1.1	1	0.6
15	0		1	0.6	0	
16	0		1	0.6	1	0.6
17	0		1	0.6	0	
18	0		1	0.6	1	0.6
19	1	0.6	0		0	
21	1	0.6	0		0	
26	0		1	0.6	0	

Table S1: Distribution of ERα/Src, ERα/PI3K and ERα/mERα data. This table shows the distribution of the number of dots/cell cfor each couple in the 175 breast tumors.

Clinical parameters	Duolink ERα/mERα				Test
	0-3<br>N=76		>3<br>N=99		
	**N**	**%**	**N**	**%**	
**Age at diagnosis (years)**					Chi-2<br>**P = 0.039**
< 50 ans	18	23.7	38	38.4	
>=50 ans	58	76.3	61	61.6	
**Menopause**					Chi-2<br>**P = 0.044**
ND	2		1		
No	20	27.0	41	41.8	
Yes	54	73.0	57	58.2	
**Tumour size (mm)**					Chi-2<br>P = 0.901
< 20 mm	30	39.5	40	40.4	
>= 20 mm	46	60.5	59	59.6	
**Histological grade (SBR)**					Chi-2<br>**P = 0.030**
1	18	23.7	14	14.1	
2	23	30.3	49	49.5	
3	35	46.1	36	36.4	
**Lymph node involvement**					Chi-2<br>P = 0.347
N0	29	38.2	47	47.5	
Micro metastasis	9	11.8	7	7.1	
Macro metastasis	38	50.0	45	45.5	
**Lympho-vascular invasion**					Chi-2<br>P = 0.937
Yes	35	46.1	45	45.5	
No	41	53.9	54	54.5	
**Estrogen receptor : % marked cells**					Chi-2<br>**P = 0.022**
ND	1		0		
< 10%	24	32.0	17	17.2	
>= 10%	51	68.0	82	82.8	
**Progesterone receptor : % marked cells**					Chi-2<br>P = 0.584
ND	1		0		
< 10%	28	37.3	33	33.3	
>= 10%	47	62.7	66	66.7	
**HER2 status**					Chi-2<br>P = 0.174
ND	7		3		
0/+/++FISH-	62	89.9	79	82.3	
++FISH+/+++	7	10.1	17	17.7	

Table S2: Distribution of clinical parameters according to groups of ERα/mERα expression. Clinical parameters (age at diagnosis, tumor size, menopausal status, lymph node involvement, SBR grading and hormonal expression) were analyzed for the 175 patients included in the TMA study. Association between clinical characteristics and the level of ERα/mERα interaction was determined using χ^2 test or Fisher's exact test.

Clinical Parameters	All N=175	
**Age at diagnosis (years)**		
< 50	56	(32.0%)
>=50	119	(68.0%)
**Menopause**		
ND	3	
No	61	(35.5%)
Yes	111	(64.5%)
**Tumor size (mm)**		
< 20 mm	70	(40.0%)
>= 20 mm	105	(60.0%)
**Histological grade (SBR)**		
1	32	(18.3%)
2	72	(41.1%)
3	71	(40.6%)
**Lymph node involvement**		
N0	76	(43.4%)
Micro metastasis	16	(9.1%)
Macro metastasis	83	(47.4%)
**Lympho-vascular invasion**		
Yes	80	(45.7%)
No	95	(54.3%)
**Estrogen receptor : % marked cells**		
ND	1	
< 10%	41	(23.6%)
>= 10%	133	(76.4%)
**Progesterone receptor : % marked cells**		
ND	1	
< 10%	61	(35.1%)
>= 10%	113	(64.9%)
**HER2 status**		
ND	10	
0/+/++FISH-	141	(85.5%)
++FISH+/+++	24	(14.5%)

Table S3: **Sample description**: **Distribution of clinical parameters.**
Clinical parameters (age at diagnosis, tumor size, menopausal status, lymph node involvement, SBR grading and hormonal expression) were analyzed for the 175 patients included in the TMA study with a cut off at 3 dots/cell.
